# Author Correction: Multivalent 9-*O*-Acetylated-sialic acid glycoclusters as potent inhibitors for SARS-CoV-2 infection

**DOI:** 10.1038/s41467-022-31290-8

**Published:** 2022-06-24

**Authors:** Simon J. L. Petitjean, Wenzhang Chen, Melanie Koehler, Ravikumar Jimmidi, Jinsung Yang, Danahe Mohammed, Blinera Juniku, Megan L. Stanifer, Steeve Boulant, Stéphane P. Vincent, David Alsteens

**Affiliations:** 1 Louvain Institute of Biomolecular Science and Technology, Université catholique de Louvain, Louvain-la-Neuve, Belgium; 2 Laboratory of Bio-Organic Chemistry (NARILIS), UNamur, Namur, Belgium; 3 Dept. of Infectious Diseases, Medical Faculty, Center for Integrative Infectious Diseases Research (CIID), University of Heidelberg, 69120 Heidelberg, Germany; 4 Department of Molecular Genetics and Microbiology, College of Medicine, University of Florida, Gainesville, USA; 5 Walloon Excellence in Life sciences and Biotechnology (WELBIO), Wavre, Belgium

**Keywords:** Nanoscale biophysics, SARS-CoV-2, Applications of AFM

Correction to: *Nature Communications* 10.1038/s41467-022-30313-8, published online 10 May 2022.

The original version of this Article contained a factual error within the discussion section, which incorrectly stated that SARS-CoV-2 expresses a hemagglutinin esterase. This has been corrected in the PDF and HTML version of the Article by the removal of the corresponding text.

The deleted text is reproduced below:

Besides the spike glycoprotein, coronaviruses, including SARS-CoV-2, express at their surface the hemagglutinin esterase (HE) that often contains an active lectin domain that mediates glycan binding in several other viruses. However, the HE lectin domains of HCoV-OC43 and HCoV-HKU1 lost their glycan binding capacity due to mutations and deletion during adaptation to human host^62^. Our result also suggests that HE might not be directly involved in 9-AcSA binding, as binding kinetics to AcSA is similar on purified S1 and at the virion level. However, as their esterase activity is maintained, HE could be involved in the deacetylation during release of viral progeny from the host cell surface and possibly also for breaking decoy interactions of the S protein with O-AcSA carrying mucins^63^.
