# Supplementary material for: Multivalent 9-O-Acetylated-sialic acid glycoclusters as potent inhibitors for SARS-CoV-2 infection
Source: Nat Commun. 2022 May 10;13:2564. doi: 10.1038/s41467-022-30313-8 (PMC9091252; doi:10.1038/s41467-022-30313-8)
Supplement: Supplementary file 1 — Supplementary Information [file 41467_2022_30313_MOESM1_ESM.pdf]

Supplementary Information for

Multivalent 9-*O*-Acetylated-sialic acid glycoclusters as potent inhibitors for SARS-CoV-2 infection.

Petitjean *et al*.

This PDF file includes:

- Supplementary Figures 1 to 6
- Supplementary Note 1: Detailed synthesis and characterization of 9-AcSA-derived multivalent SA glycoclusters
- Supplementary Note 2: Synthesis and characterization of biotinylated sialic acids

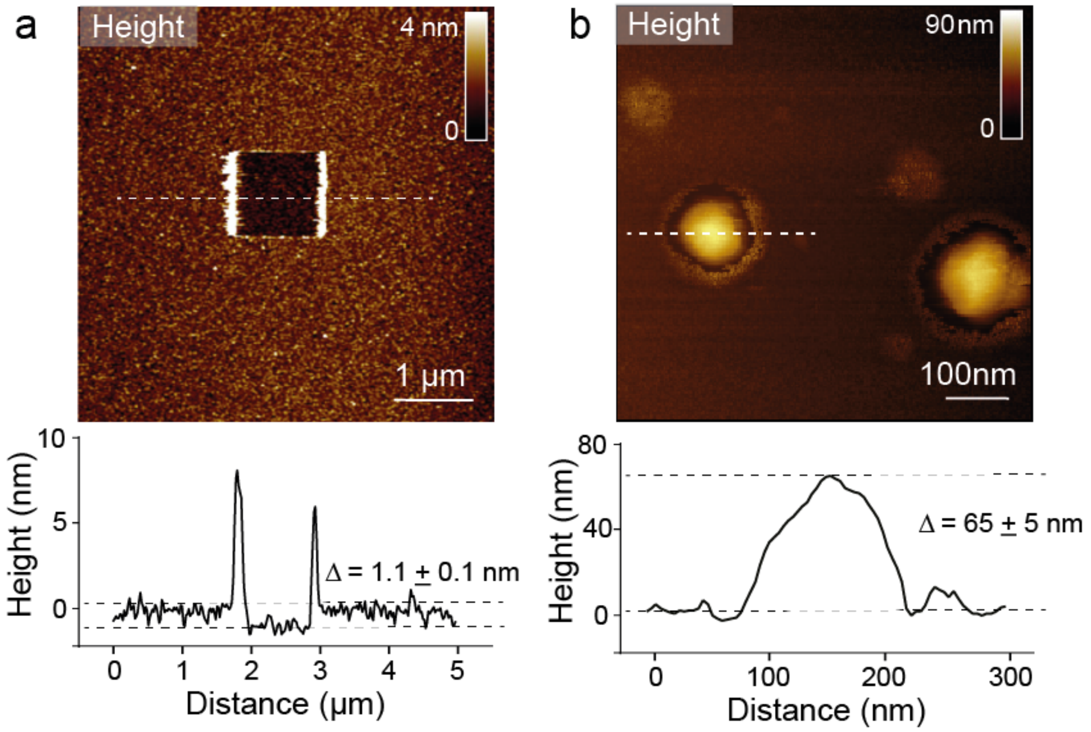

Supplementary Figure 1| Validation of surface immobilization and characterization of non-replicating SARS-CoV-2 particles. (**a**) AFM topography image of a 9Ac-SA coated surface after scanning a 1 x 1 µm area at high forces (~ 18 nN) to remove the attached biomolecules (referred to as "scratching" experiment). Below: Cross-section taken along the white dashed line, showing an accumulation of biological materials on the sides of the square. The biomolecule-free surface of inside the square was 1.1 ± 0.1 nm lower than the surrounding biomolecule-coated surface, providing an estimate of the thickness of 9-Ac-SA deposited layer. The experiment was repeared 2 times independently. (**b**) AFM height image of non-replicating SARS-CoV-2 particles deposited on freshly cleaved mica substrate. The cross-section taken along the white dashed line is shown below. The height of the particles amounts to 65 ± 5nm. The experiment was repeared 2 times independently.

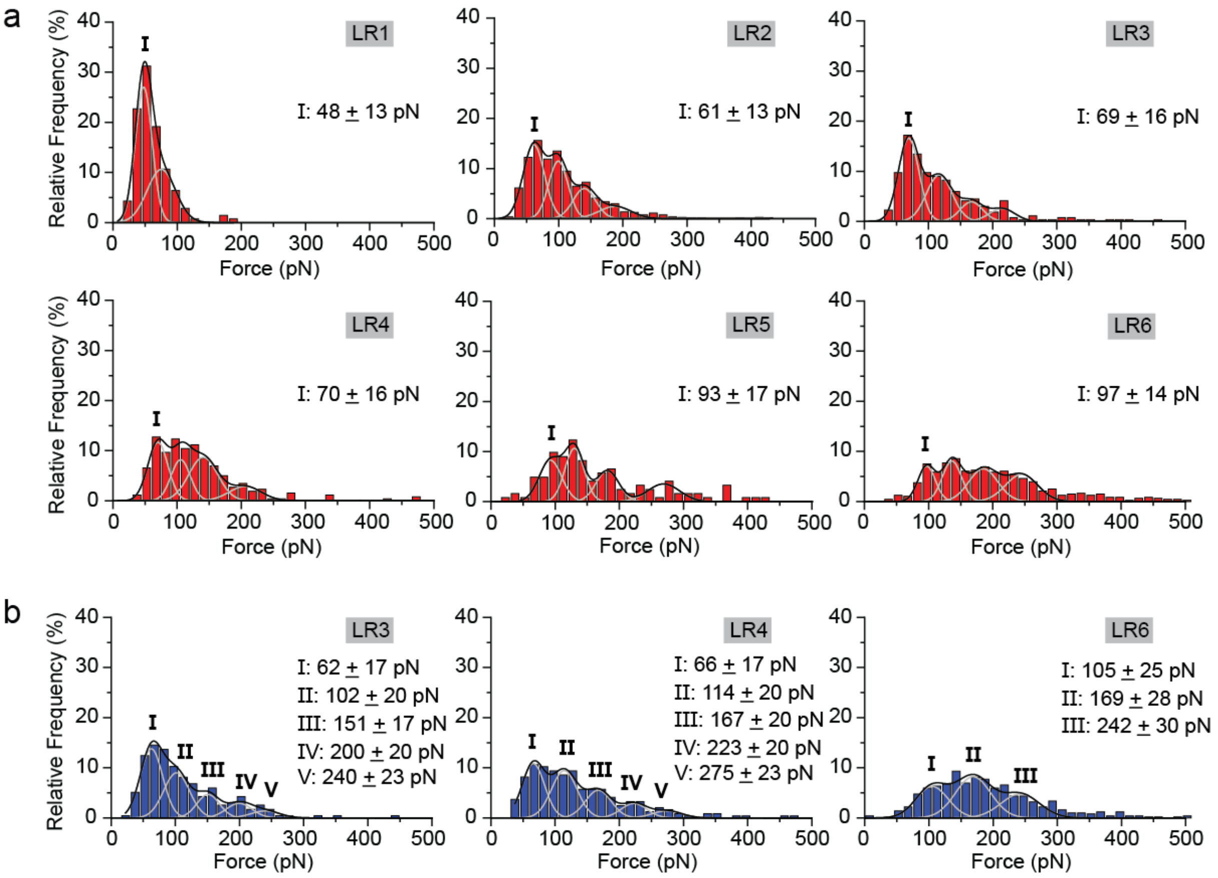

Supplementary Figure 2| Probing S1 (a) and SARS-CoV-2 (b) binding to 9Ac-SA model surfaces. (**a**) S1 – 9AcSA interaction: Force and LR were extracted from force-distance curves and sorted in narrow LR ranges (LR1-LR6). The rupture forces for each LR range were plotted as histograms and fitted with multipeak Gaussian fits. The maximum force of the first peak (I, monovalent interaction) is indicated. N = 2330 from 6 independent experiments. (**b**) SARS-CoV-2 – 9AcSA interaction: Force and LR were extracted from force-distance curves and sorted in three LR ranges (LR3, LR4, LR6 – corresponding to a). The rupture forces for each LR range were plotted as histograms and fitted with multipeak Gaussian fits. The maxima of all force peaks (I – V, multivalent interactions) are indicated. N = 511 from 5 independent experiments.

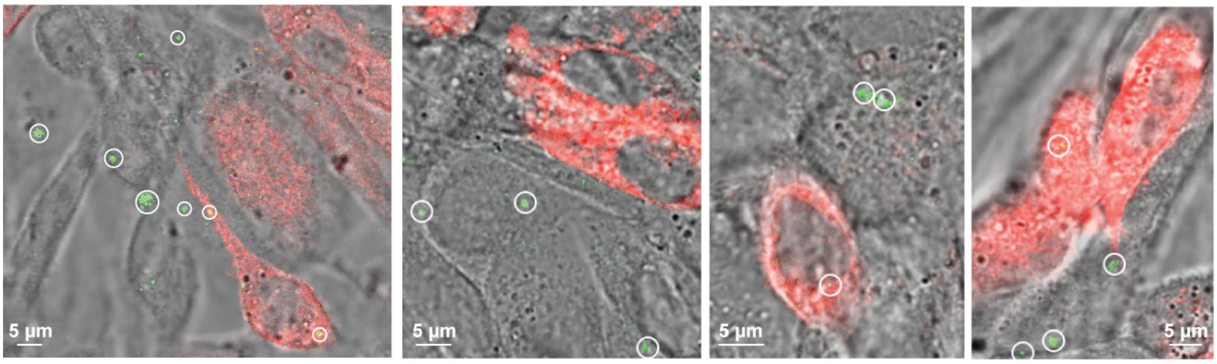

Supplementary Figure 3| Virus binding assay. Confocal microscopy of a coculture of SA-expressing CHO and fluorescently labelled Lec2 cells, deficient in SA expression (mCherry, red) incubated with UV-inactivated SARS-CoV-2 virions (fluorescently labelled with an Atto488-NHS ester dye, green and highlighted in white circle). Virions mainly bind to CHO cells, highlighting the role of SA in cellular adhesion.

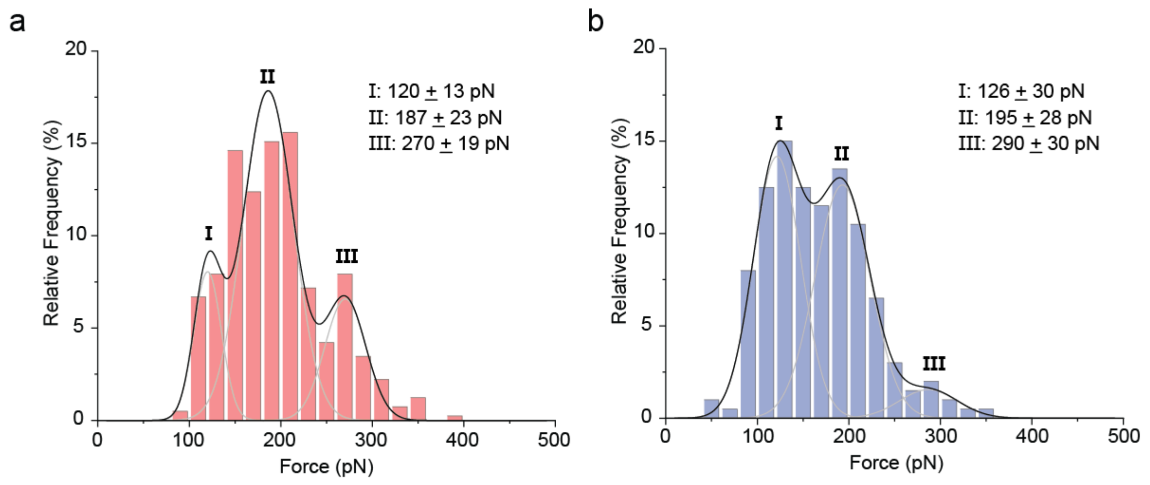

Supplementary Figure 4| Probing S1 and SARS-CoV-2 binding to living cells. Force and LR of probing living cells with (**a**) S1-tip or (**b**) SARS-CoV-2-tip were extracted from force-time curves and plotted as histograms and further fitted with multipeak Gaussian fits. The maxima of all force peaks (I – III, multivalent interactions) are indicated. N = 644 curves for S1 (a) and N = 199 curves for SARS-CoV-2 (b) from 3 independent experiments.

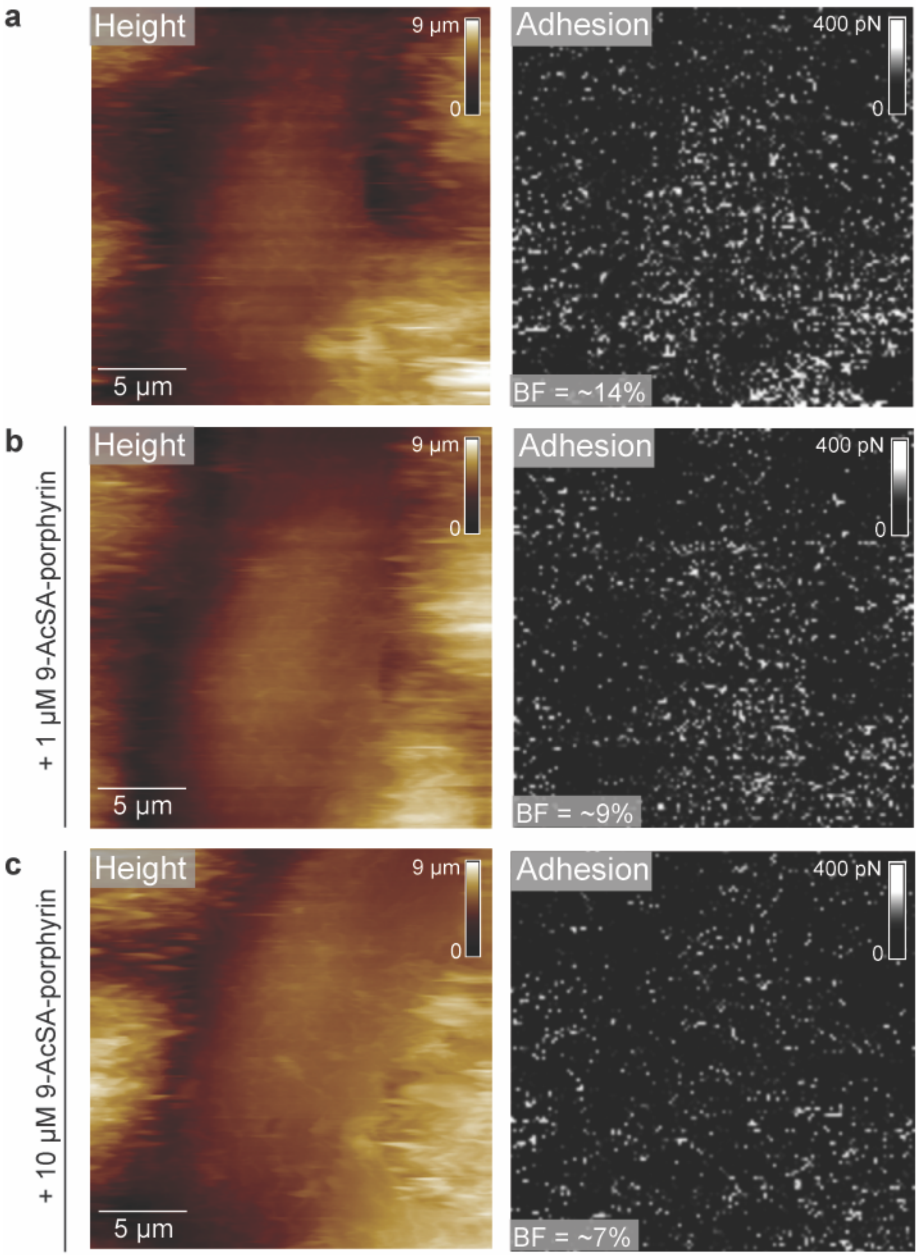

Supplementary Figure 5| Characterization of 9-AcSA-derived porphyrin as a binding inhibitor on living cells. FD-based AFM was used to image the height and to obtain adhesion forces in the corresponding area of CHO cells probed with SARS-CoV-2 virions first in the absence of 9-AcSA-porphyrin (**a**), and subsequently after injection of 1 µM (**b**) and 10 µM (**c**) 9-AcSA-porphyrin. A significant reduction in adhesion events can be observed following addition of the SA glycocluster. All AFM images were acquired using an oscillation frequency of 0.25 kHz and amplitude of 750 nm, under cell culture conditions. Experiments were repeated 3 times.

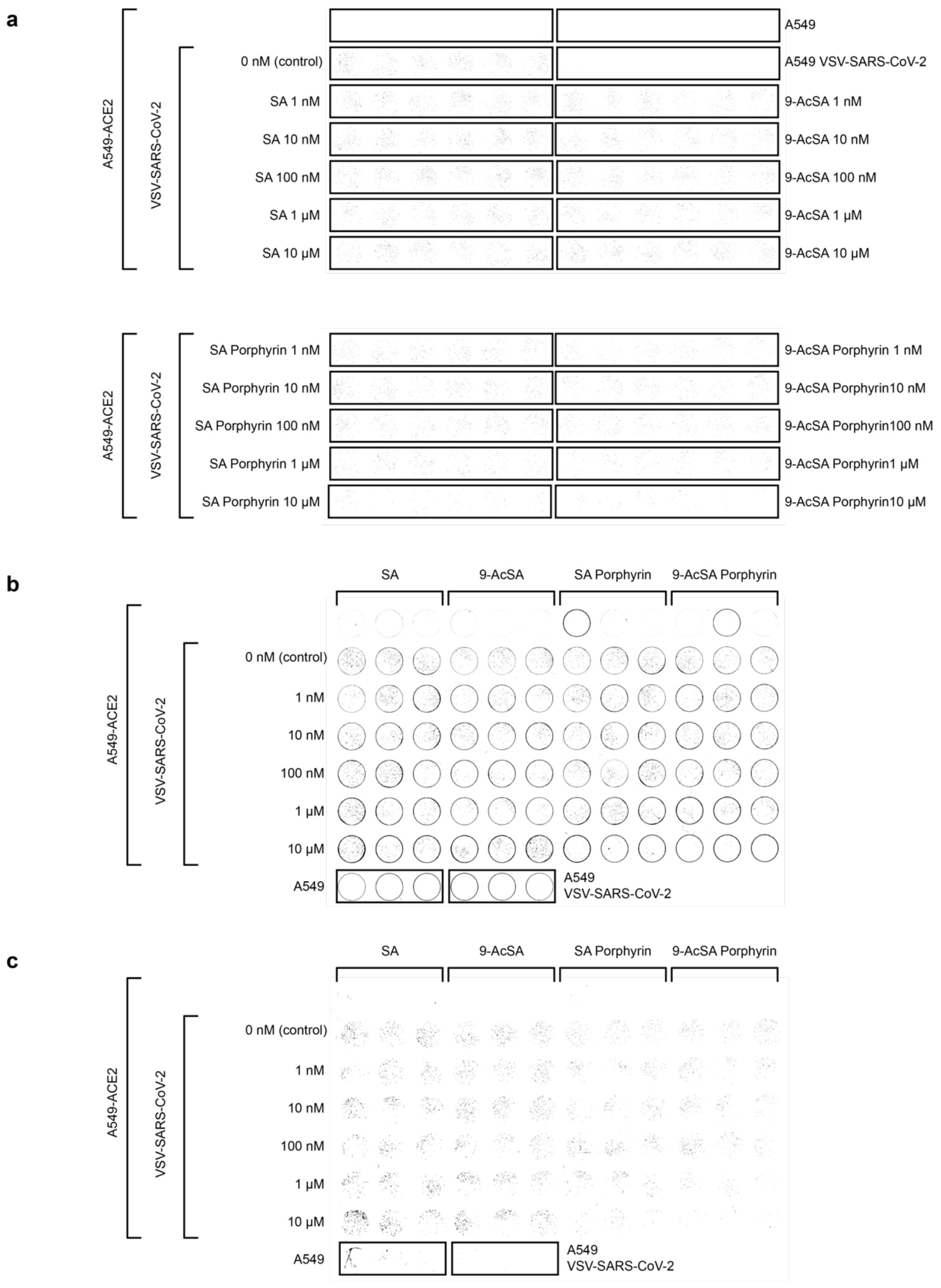

Supplementary Figure 6| Images of infected cells by VSV-SARS-CoV-2 and incubated with various concentration of SA, 9-AcSA, SA/9-AcSA-porphyrin. (**a,b,c**) Infection assays where the grey dots show the infected cells. a,b and c are three independent replicates. Images are acquired with a resolution of 10 µm.

Supplementary Note 1: Detailed synthesis and characterization of 9-AcSA-derived multivalent SA glycoclusters

General methods and materials

The solvents used for chromatography were purchased in industrial grade and further distilled before their use. Reagents and chemicals were purchased from Sigma-Aldrich or Acros at ACS grade and were used without purification. All reactions were monitored by thin-layer chromatography (TLC) carried out on Merck aluminum roll silica gel 60-F_{254} using $KMnO_4$ and a phosphomolybdic acid solution as revelators. Merck silica gel (60, particle size 40-63 µm) was employed for flash column chromatography. NMR spectra were recorded on a JEOL ECX 400 or 500 with solvent peaks as reference. The compounds were characterized by ^1H and ^{13}C NMR as well as by ^1H-^1H and ^1H-^{13}C correlation experiments. The abbreviations used to define the multiplicities are: s = singlet, d = doublet, t = triplet, q = quadruplet, m = multiplet and br = broad. Chemical shifts (δ) are reported in ppm and referenced indirectly to the residual solvent signals. High resolution mass spectra (HRMS) were carried out on the Bruker maXis Impact QTOF mass spectrometer and Bruker MicroTOF-Q II XL spectrometer.

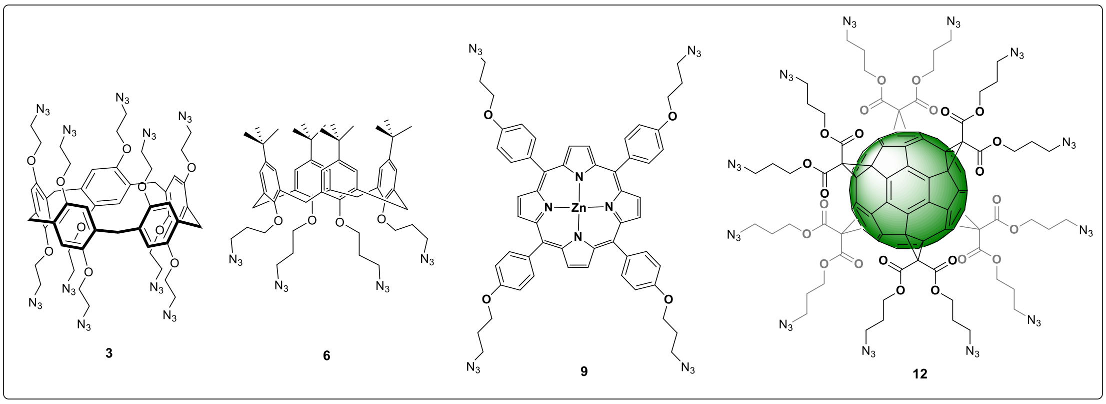

Figure S1. Structures of compound **3**, **6**, **9** and **12**

The four compounds **3**[1], **6**[2], **9**[2,3], and **12**[4] were prepared according to known procedures.

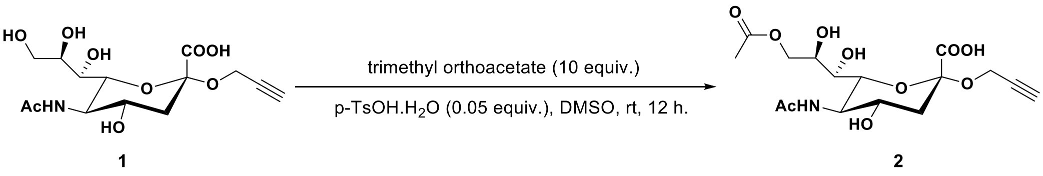

Scheme S1. Synthesis of compound **2**

Prop-2-ynyl 5-acetamido-9-*O*-acetyl-3,5-dideoxy-D-glycero-α-D-galacto-2-onulopyranose (2)

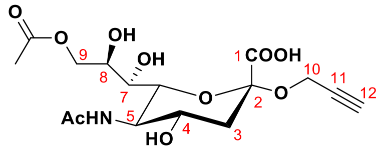

The title compound was prepared following a literature procedure.[5] To a solution of **1**[6] (380 mg, 1.1 mmol, 1 equiv.) and trimethyl orthoacetate (1.4 mL, 11 mmol, 10 eq.) in dry DMSO (4 mL) was added *p*-toluenesulfonic acid monohydrate (10 mg, 0.05 mmol, 0.05 equiv.). The solution was stirred at room temperature for 12 h. Then, DCM (20 mL) was added to precipitate the crude product. After a slow cotton filtration, the solid on the filtrate was redissolved in methanol and transferred to evaporate the solvent. The residue was purified by silica gel chromatography using DCM/MeOH (20:3) to afford the desired compound as a white solid (167 mg, 0.429 mmol, 39% yield).

^{1}H NMR (500 MHz, CD_3OD) δ: 4.42–4.35 (m, 2H, H-9a, H-10a), 4.17–4.03 (m, 3H, H-10b, H-9b, H-8), 3.74–3.65 (m, 2H, H-4, H-5), 3.57–3.54 (m, 1H, H-6), 3.49 (dd, J = 9.2, 1.9 Hz, 1H, H-7), 2.83 (dd, J = 12.3, 4.4 Hz, 1H, H-3a), 2.75 (t, J = 2.4 Hz, 1H, H-13), 2.06 (s, 3H, $OCOCH_3$), 2.03 (s, 3H, $NHCOCH_3$), 1.60 (t, J = 11.7 Hz, 1H, H-3b).

^{13}C NMR (126 MHz, CD_3OD) δ 175.6 (C=O, NAc), 173.5 (C-1), 173.1 (C=O, 9-O-Ac), 101.6 (C-2), 80.6 (C-11), 75.0 (C-12), 74.1 (C-6), 70.6 (C-8), 70.5 (C-7), 69.2 (C-4), 67.2 (C-9), 54.0 (C-5), 53.1 (C-10), 42.2 (C-3), 22.7 ($NCOCH_3$), 20.8 ($OCOCH_3$).

HRMS (TOF-MS-ESI$^+$, m/z): calculated for $C_{16}H_{24}NO_{10}$ $[M+H]^+$ 390.1395; found 390.1392.

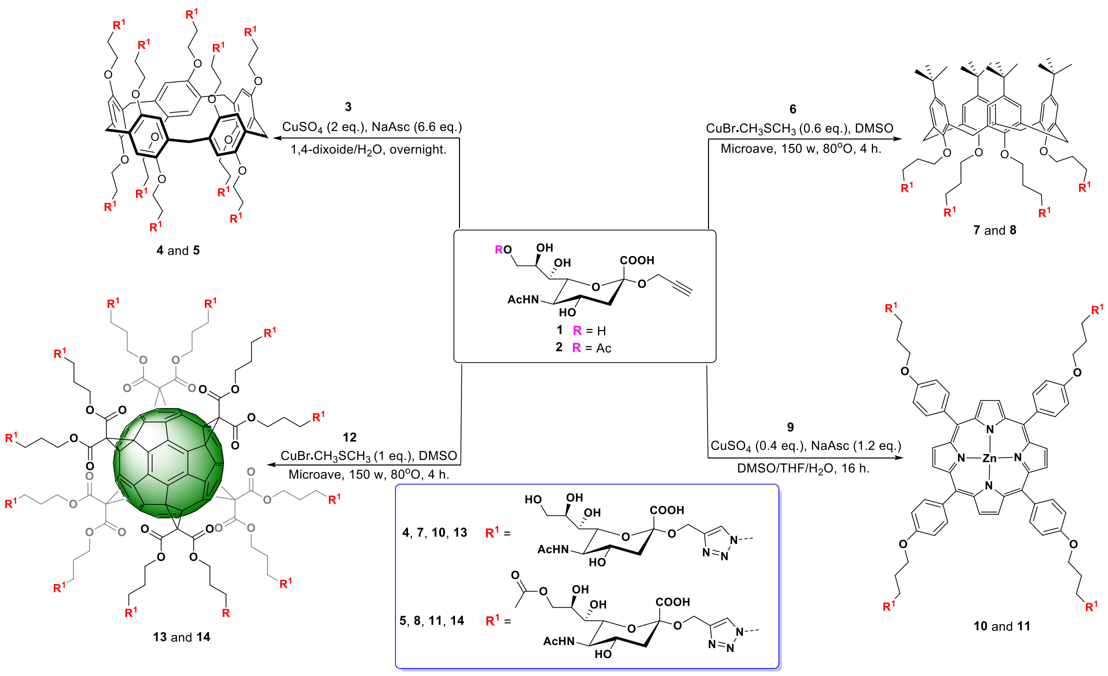

Scheme S2. Synthesis of targeted multimeric sialic acids ligand by CuAAC reactions

Compound 4

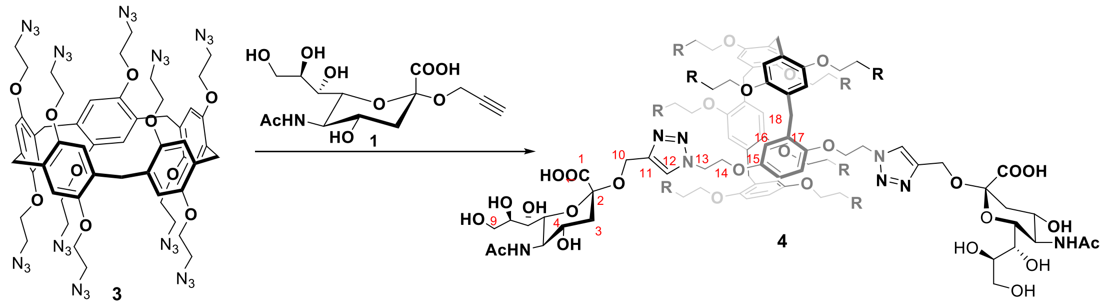

To a solution of **3**[1] (21 mg, 0.01 mmol, 1 equiv.) and **1**[6] (70 mg, 0.20 mmol, 12 equiv.) in 1,4-dioxane (2 mL) was added a freshly prepared solution of $CuSO_4$ (3.2 mg, 0.02 mmol, 2 equiv.) and NaAsc (13 mg, 0.066 mmol, 6.6 equiv.) in H_2O (1 mL) under argon atmosphere. The reaction mixture was vigorously stirred overnight at room temperature. Then acetone (40 mL) was added to precipitate the crude product. After centrifugation, the crude was dissolved in water (2 mL) and treated with Quadrasil MP (40 mg) to remove the residual copper ions. After filtration through a 45 µm sterile filter, the filtrate was passed through a Sephadex® G-25 column and eluted with water. The fractions that could not migrate by TLC elution with DCM/MeOH (1:0.3, $KMnO_4$ staining) were collected. The combined fractions were lyophilized to afford a white solid (59 mg, 0.0124 mmol, 74% yield).

^1H NMR (500 MHz, DMSO-d$_6$) δ 8.17–8.06 (m, 10H, 10 x H-12), 6.57 (s, 10H, 10 x H-16), 4.77–4.13 (m, 60H, 10 x H-10, 10 x H-13, 10 x H-14), 3.61–3.31 (m, 80H, 10 x H-4, 10 x H-5, 10 x H-6, 10 x H-7, 10 x H-8, 10 x H-9, 10 x H-18), 2.63 (s, 10H, 10 x H-3a), 1.87 (s, 30H, 10 x NCOCH$_3$), 1.43 (s, 10H, 10 x H-3b).

^{13}C NMR (126 MHz DMSO-d$_6$) δ 173.9 (C=O, NCOCH$_3$), 172.0 (C-1), 149.7 (Cq, C-15), 145.2 (Cq, C-11), 129.6 (Cq, C-17), 125.2 (C-12), 116.1 (C-16), 100.5 (C-2), 73.5 (C-6), 72.1 (C-8), 69.3 (C-7), 68.1 (C-4, C-14), 63.6 (C-9), 57.8 (C-10), 53.2 (C-5), 50.8 (C-13), 41.6 (C-3), 29.0 (C-18), 23.2 (NCOCH$_3$).
HRMS (TOF-MS-ESI$^-$, m/z): calculated for $C_{195}H_{267}N_{40}O_{100}$ $[M-3H]^{3-}$ 1590.2365; found 1590.2262.

Compound 5

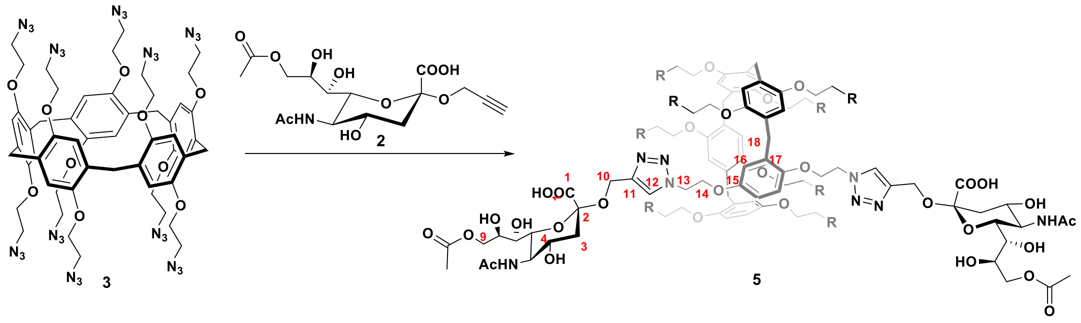

To a solution of **3**[1] (13 mg, 0.01 mmol, 1 equiv.) and **2** (47 mg, 0.12 mmol, 12 equiv.) in 1,4-dioxane (1.2 mL) was added a freshly prepared solution of $CuSO_4$ (3.2 mg, 0.02 mmol, 2 equiv.) and NaAsc (13 mg, 0.066 mmol, 6.6 equiv.) in H_2O (0.6 mL) under argon atmosphere. The reaction mixture was vigorously stirred overnight at room temperature. Then, acetone (20 mL) was added to precipitate the crude product. After centrifugation, the crude was dissolved in water (2 mL) and treated with Quadrasil MP (40 mg) to remove the residual copper ions. After filtration through a 45 µm sterile filter, the filtrate was passed through a Sephadex®G-25 column and eluted with water. The fractions that could not migrate by TLC elution with DCM/MeOH (1:0.3) and $KMnO_4$ staining were collected. The combined fractions were lyophilized to afford a white solid (32 mg, 0.0062 mmol, 62% yield).

^{1}H NMR (500 MHz, CD_3OD) δ 8.16 (br, 10H, 10 x H-12), 6.62 (br, 10H, 10 x H-16), 4.69 (br, 30H, 10 x H-10b, 10 x H-13), 4.37 (br, 20H, 10 x H-9), 4.09 (br, 30H, 10 x H-8, 10 x H-14), 3.76 (br, 40H, 10 x H-4, 10 x H-5, 10 x H-6, 10 x H-18), 3.53 (br, 10H, 10 x H-7), 2.83 (br, 10H, 10 x H-3a), 2.03 (br, 60H, 10 x $OCOCH_3$, 10 x $NCOCH_3$), 1.65 (br, 10H, 10 x H-13b).

^{13}C NMR (126 MHz, CD_3OD) δ 175.3 (C=O, $NCOCH_3$), 173.9 (C-1), 173.3 (C=O, 9-O-Ac), 151.0 (Cq, C-15), 146.3 (Cq, C-11), 130.1 (Cq, C-17), 126.0 (C-12), 117.1 (C-16), 101.7 (C-2), 74.3 (C-6), 70.6 (C-8), 69.3 (C-7), 68.6 (C-4, C-14), 67.3 (C-9), 58.9 (C-10), 54.0 (C-5), 51.4 (C-13), 42.1 (C-3), 31.0 (C-18), 22.9 ($NCOCH_3$), 21.0 ($OCOCH_3$).

HRMS (TOF-MS-ESI$^+$, m/z): calculated for $C_{215}H_{293}N_{40}O_{110}$ $[M+3H]^{3+}$ 1732.2869; found 1732.2863.

Compound 7

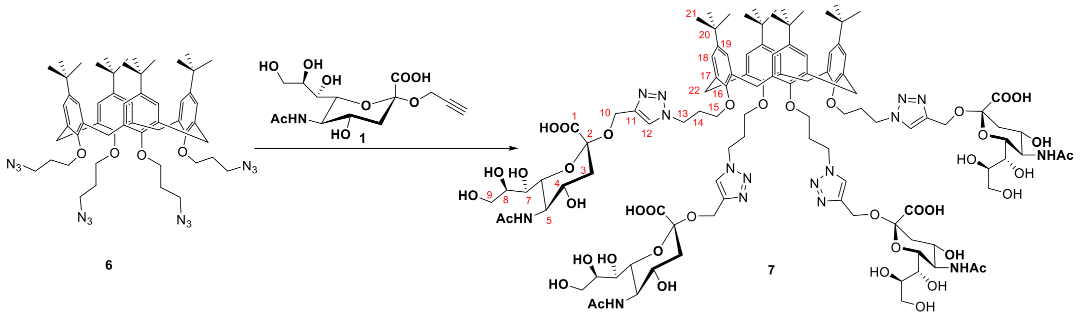

To a solution of **6**[2] (36 mg, 0.03 mmol, 1 equiv.) and **1**[6] (46 mg, 0.132 mmol, 4.4 equiv.) in dry DMSO (1 mL) was added $CuBr{\cdot}CH_3SCH_3$ (3.7 mg, 0.018 mmol, 0.6 equiv.) under argon atmosphere. After 4 h under microwave irradiations at 80°C, the crude was cooled down and precipitated by adding DCM (30 mL). The precipitate was then dissolved in water (2 mL) and treated with Quadrasil®MP (70 mg) to remove the residual copper ions. After filtration through a 45 µm sterile filter, the filtrate was passed through a Sephadex® G-15 column and eluted with water. The fractions that could not migrate by TLC elution with DCM/MeOH (1:0.3) and $KMnO_4$ staining were collected. The combined fractions were lyophilized to afford a white solid (70 mg, 0.0276 mmol, 92% yield).

^{1}H NMR (500 MHz, CD_3OD) δ 8.03 (s, 4H, 4 x H-12), 6.82 (s, 8H, 8 x H-18), 4.62 (br, 8H, 8 x H-13), 4.35 (br, 4H, 4 x H-22a), 3.89–3.58 (m, 52H, 4 x H-4, 4 x H-5, 4 x H-6, 4 x H-7, 4 x H-8, 4 x H-9, 4 x H-15, 16 x OH), 3.13 (br, 4H, 4 x H-22b), 2.82 (br, 4H, 4 x H-3a), 2.52 (br, 8H, 4 x H-14), 2.02 (s, 12H, 4 x $NCOCH_3$), 1.66 (br, 4H, 4 x H-3b), 1.08 (s, 36H, 12 x H-21).

^{13}C NMR (126 MHz, CD_3OD) δ 175.3 (C=O, $NCOCH_3$), 173.9 (C-1), 154.2 (Cq, C-16), 146.1 (Cq, C-11, C-19), 134.9 (Cq, C-17), 126.4 (C-18), 125.6 (C-12), 101.3 (C-2), 74.50 (C-6), 73.0 (C-15), 70.1 (C-7, C-8), 69.2 (C-4), 64.3 (C-9), 58.4 (C-10), 53.9 (C-5), 48.5 (C-13), 41.9 (C-3), 34.7 (Cq, C-20), 32.0 (C-14, C-21, C-22), 22.9 ($NCOCH_3$).

HRMS (TOF-MS-ESI$^+$, m/z): after deconvolution calculated for $C_{120}H_{170}N_{16}O_{44}$ [M] 2369.0978; found 2369.0877.

Compound 8

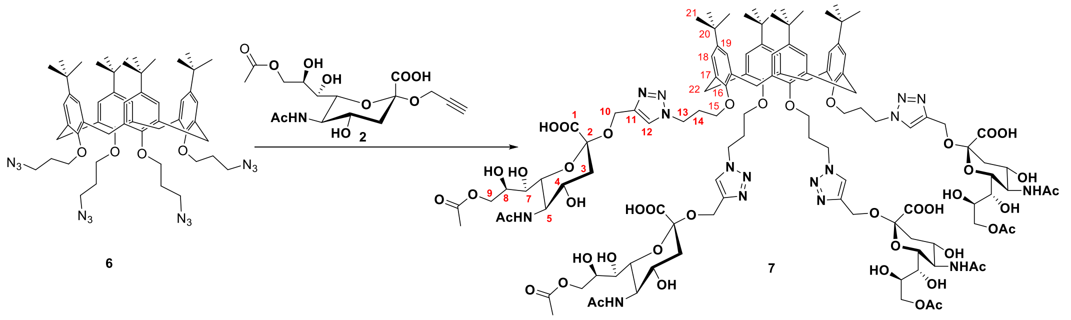

To a solution of **6**[2] (37 mg, 0.03 mmol, 1 equiv.) and **2** (51 mg, 0.132 mmol, 4.4 equiv.) in dry DMSO (1 mL) was added $CuBr{\cdot}CH_3SCH_3$ (3.7 mg, 0.018 mmol, 0.6 equiv.) under argon atmosphere. After 4 h under microwave irradiations at 80°C, the crude was precipitated by adding DCM (30 mL). The precipitate was then dissolved in water (2 mL) and treated with Quadrasil®MP (70 mg) to remove the residual copper ions. After filtration through a 45 µm sterile filter, the filtrate was passed through a Sephadex® G-15 column and eluted with water. The fractions that could not migrate by TLC elution with DCM/MeOH (1:0.3, $KMnO_4$ staining) were collected. The combined fractions were lyophilized to afford a white solid (62 mg, 0.0244 mmol, 81% yield).

^1H NMR (500 MHz, CD_3OD) δ 8.21–8.06 (m, 4H, 4 x H-12), 6.82 (s, 8H, 8 x H-18), 4.63 (br, 8H, 4 x H-13), 4.37 (s, 8H, 4 x H-9a, 4 x H-22a), 4.13 (br, 8H, 4 x H-8, 4 x H-9b), 3.88–3.52 (m, 24H, 4 x H-4, 4 x H-5, 4 x H-6, 4 x H-7, 4 x H-15), 3.13 (s, 4H, 4 x H-22b), 2.79 (br, 4H, 4 x H-3a), 2.53 (br, 8H, 4 x H-14), 2.02 (br, 24H, 4 x $NCOCH_3$, 4 x $OCOCH_3$), 1.70 (br, 4H, 4 x H-3b), 1.33 (s, 6H, 2 x H-21), 1.08 (s, 24H, 8 x H-21), 0.85 (s, 6H, 2 x H-21).

^{13}C NMR (126 MHz, CD_3OD) δ 175.3 (C=O, $NCOCH_3$), 173.1 (C=O, C-1, 9-O-Ac), 154.2 (Cq, C-16), 146.4 (Cq, C-11), 146.0 (Cq, C-19), 134.9 (Cq, C-17), 126.4 (Cq, C-12, C-18), 100.8 (C-2), 74.4

(C-6), 72.9 (C-15), 70.5 (C-8), 70.4 (C-7), 69.1 (C-4), 67.4 (C-9), 58.7 (C-10), 53.8 (C-5), 42.0 (C-3), 34.7 (Cq, C-20), 32.0 (C-14, C-21, C-22), 22.75 ($OCOCH_3$), 20.9 ($OCOCH_3$).

HRMS (TOF-MS-ESI$^+$, m/z): after deconvolution calculated for $C_{120}H_{170}N_{16}O_{44}$ [M] 2537.1400; found 2537.1298.

Compound 10

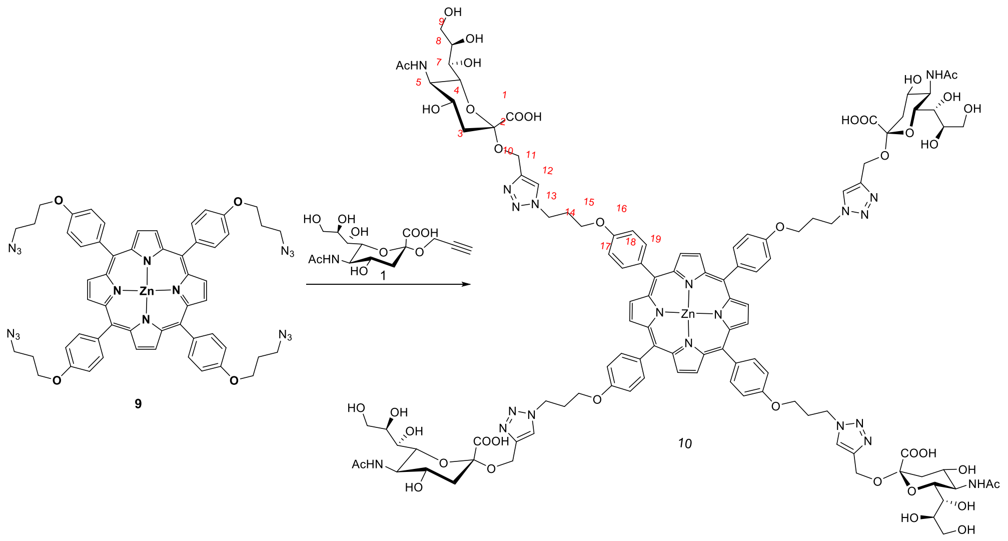

To a solution of **9**[2,3] (40 mg, 0.037 mmol µmol, 1 equiv.) and **1**[6] (57 mg, 0.164 mmol, 4.4 equiv,) in THF/DMSO (1.5 mL, 2:3) was added a freshly prepared solution of $CuSO_4$ (2.36 mg, 0.0148 mmol, 0.4 equiv.) and NaAsc (8.7 mg, 0.0439 mmol, 1.2 equiv.) in H_2O (0.3 mL) under argon atmosphere. The reaction mixture was vigorously stirred overnight at room temperature. Then, acetone (30 mL) was added to precipitate the crude product. The crude was then dissolved in water (2 mL) and treated with Quadrasil®MP (80 mg) to remove the residual copper ions. After filtration through a 45 µm sterile filter, the filtrate was passed through a Sephadex® G-15 column and eluted with water. The fractions that could not migrate by TLC elution with DCM/MeOH (1:0.3, $KMnO_4$ staining) were collected. The combined fractions were lyophilized to afford a dark green solid (56 mg, 0.0227 mmol, 61% yield).

^{1}H NMR (400 MHz, D_2O/DMSO-d_6) δ 9.07 (br, 8H, 8 x H-$_{Porph}$), 8.32 (br, 12H, 4 x H-12, 8 x H-18), 7.51 (br, 8H, 8 x H-17), 3.74 (br, 28H, 4 x H-4, 4 x H-5, 4 x H-6, 4 x H-7, 4 x H-8, 4 x H-9), 2.78 (br, 12H, 4 x H-14, 4 x H-3a), 2.05 (br, 16H, 4 x H-3b, 4 x $NCOCH_3$).

^{13}C NMR (101 MHz, D_2O/DMSO-d_6) δ 176.3 (C=O, $NCOCH_3$), 173.9 (C-1), 159.5 (C-16), 151.3 (Cq, C-19), 146.6 (Cq, C-11), 137.2 (C-18), 135.5 (Cq, C-$_{Porph}$), 133.3 (C-$_{Porph}$), 127.0 (C-12), 121.5

(Cq, C-Porph), 114.5 (C-17), 101.9 (C-2), 74.3 (C-6), 73.1 (C-8), 69.7 (C-4, C-7), 67.0 (C-15), 64.1 (C-9), 59.1 (C-10), 53.4 (C-5), 49.4 (C-13), 41.8 (C-3), 30.9 (C-14), 23.6 ($NCOCH_3$).

HRMS (TOF-MS-ESI$^+$, m/z): calculated for $C_{112}H_{134}N_{20}O_{40}Zn$ $[M+2H]^{2+}$ 1232.4186; found 1232.4390.

Compound 11

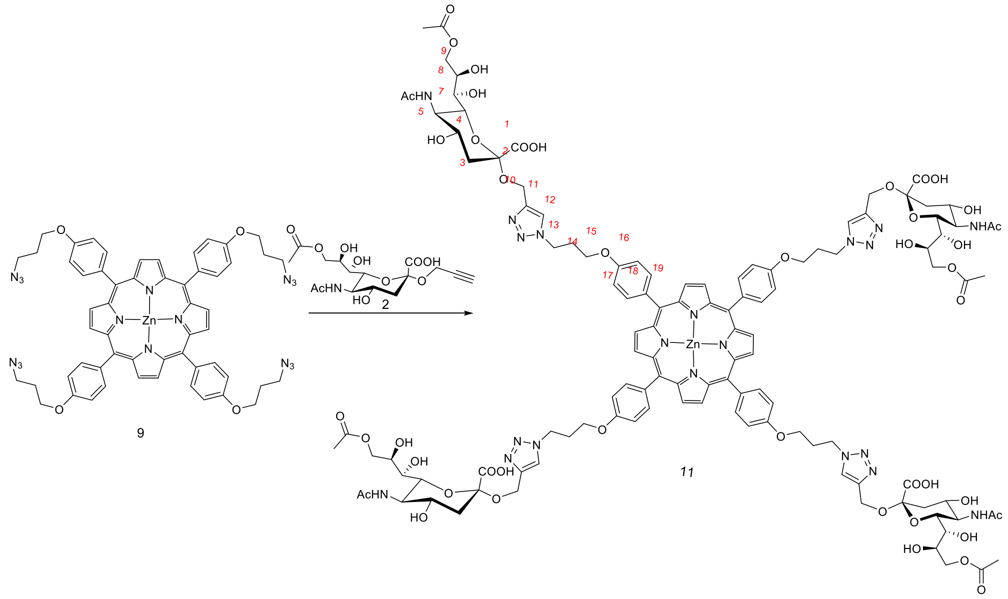

To a solution of **9**[2,3] (40 mg, 0.037 mmol µmol, 1 equiv.) and **7** (63 mg, 0.162 mmol, 4.4 equiv,) in THF/DMSO (1.8 mL, 1:1) was added a freshly prepared solution of $CuSO_4$ (2.36 mg, 0.0148 mmol, 0.4 equiv.) and NaAsc (8.7 mg, 0.0439 mmol, 1.2 equiv.) in H_2O (0.3 mL) under argon atmosphere. The reaction mixture was vigorously stirred at room temperature overnight. Then, acetone (30 mL) was added to precipitate the crude product. The crude was then dissolved in water (2 mL) and treated with Quadrasil®MP (80 mg) to remove the residual copper ions. After filtration through a 45 µm sterile filter, the filtrate was passed through a Sephadex® G-15 column and eluted with water. The fractions that could not migrate by TLC elution with DCM/MeOH (1:0.3) were collected. The combined fractions were lyophilized to afford a dark green solid (82 mg, 0.0323 mmol, 87% yield).

^1H NMR (500 MHz, DMSO-d_6) δ 8.60 (br, 8H, 8 x H-Porph), 8.11 (br, 4 x H-12), 7.72 (br, 8H, 8 x H-18), 6.89 (br, 8H, 8 x H-17), 4.18–4.04 (m, 56H, 4 x H-4, 4 x H-5, 4 x H-8, 4 x H-9, 4 x H-10, 4 x H-13, 4 x H-15, 12 x OH, 4 x NH), 3.46–3.33 (m, 8H, 4 x H-6, 4 x H-7), 2.26 (8H, 4 x H-14), 1.88 (br, 28H, 4 x $NCOCH_3$, 4 x $OCOCH_3$, 4 x H-3b).

^{13}C NMR (126 MHz, DMSO-d$_6$) δ 174.0 (C=O, NCOCH$_3$), 172.3 (C=O, C-1, 9-O-Ac), 158.3 (Cq, C-16), 150.3 (Cq, C-19), 135.7 (C-18), 132.2 (C-$_{Porph}$), 120.7 (Cq, C-$_{Porph}$), 113.2 (C-17), 73.1 (C-6), 69.6 (C-8), 68.0 (C-4, C-7), 67.0 (C-9), 65.2 (C-15), 58.1 (C-10), 53.1 (C-5), 47.8 (C-13), 41.8 (C-3), 30.1 (C-14), 23.1 (NCOCH$_3$), 21.3 (OCOCH$_3$).

HRMS (TOF-MS-ESI$^+$, m/z): calculated for $C_{120}H_{140}N_{20}O_{44}Zn$ $[M+2H]^{2+}$ 1313.4228; found 1313.3966.

Compound 13

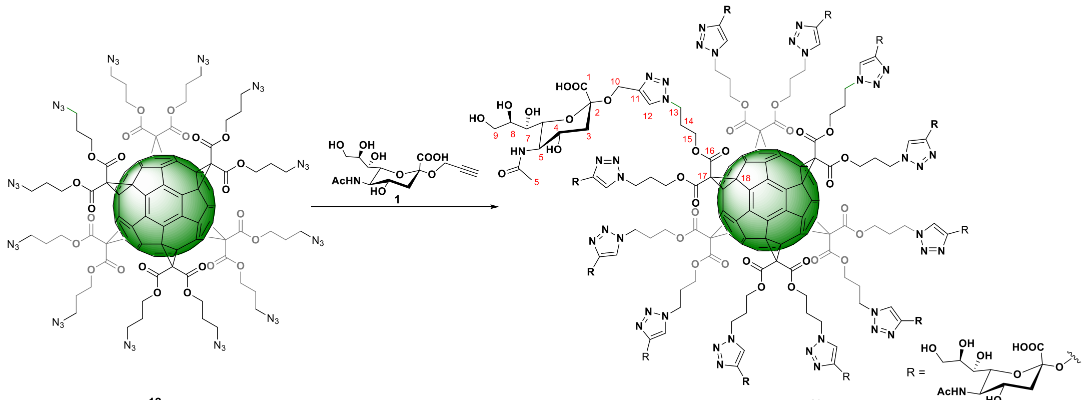

12 13

To a solution of **12**[4] (40 mg, 0.0172 mmol, 1 equiv.) and **1**[6] (95 mg, 0.274 mmol, 16 equiv.) in DMSO (1.4 mL) was added CuBr·CH$_3$SCH$_3$ (3.5 mg, 0.017 mmol, 1 equiv.) under argon atmosphere. After 4 h of microwave irradiation at 80°C, the solution was cooled down and precipitated with DCM (30 mL), and centrifuged. The crude was redissolved in water (2 mL) and treated with Quadrasil®MP (80 mg) to remove the residual copper ions. After filtration through a 45 µm sterile filter, the filtrate was passed through a Sephadex™G-25 column and eluted with water. The fractions that could not migrate by TLC elution with DCM/MeOH were collected. The combined fractions were lyophilized to afford an orange solid (89 mg, 0.0137 mmol, 80% yield).

^1H NMR (400 MHz, DMSO-d$_6$) δ 8.07–7.97 (m, 12H, 12 x H-12), 4.67 (s, 12H, 12 x H-10a), 4.40 (s, 36H, 12 x H-10b, 12 x H-15), 4.15 (br, 84H, 12 x H-12, 48 x OH, 12 x NH), 3.69–3.29 (m, 84H, 12 x H-4, 12 x H-5, 12 x H-6, 12 x H-7, 12 x H-8, 12 x H-9), 2.67 (s, 12H, 12 x H-3a), 2.15 (s, 24H, 12 x H-14), 1.86 (s, 36H, 12 x NAc), 1.36 (m, 12H, 12 x H-3b).

^{13}C NMR (101 MHz, DMSO-d$_6$) δ 174.0 (C=O, NCOCH$_3$), 171.9 (C=O, C-1), 164.0 (Cq, C-16), 145.6, 141.5 (C-11, C$_{sp2}$), 125.2 (C-12), 100.7 (C-2), 73.4 (C-6), 72.1, 69.4, 68.2 (C-18, C-8, C-7, C-4),

65.1 (C-15), 63.6 (C-9), 57.8 (C-10), 53.3 (C-5), 47.2 (C-17, C-13), 41.9 (C-3), 29.4 (C-14), 23.2 ($NCOCH_3$).

HRMS (ESI+-MS, m/z): calculated for $C_{282}H_{328}N_{48}O_{132}$ $[M+4H]^{4+}$ 1623.2468; found 1623.2138.

Compound 14

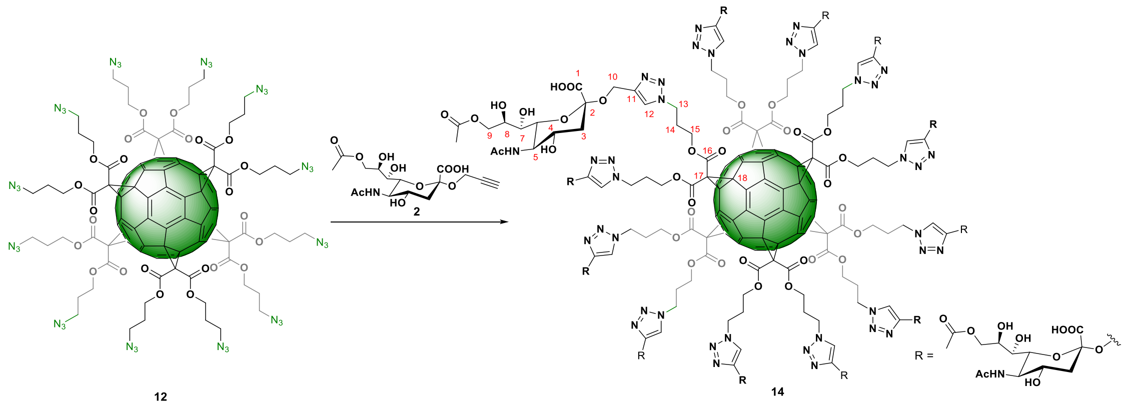

To a solution of **12**[4] (22 mg, 0.0096 mmol, 1 equiv.) and **2** (60 mg, 0.154 mmol, 16 equiv.) in DMSO (0.8 mL) was added $CuBr{\cdot}CH_3SCH_3$ (2 mg, 0.0096 mmol, 1 equiv.) under argon atmosphere. After 4 h of microwave irradiation at 80°C, the solution was cooled down and precipitated with DCM (30 mL), and centrifuged. The crude was redissolved in water (2 mL) and treated with Quadrasil®MP (60 mg) to remove the residual copper ions. After filtration through a 45 µm sterile filter, the filtrate was passed through a Sephadex™G-25 column and eluted with water. The fractions that could not migrate by TLC elution with DCM/MeOH (1:0.3, $KMnO_4$ staining) were collected. The combined fractions were lyophilized to afford an orange solid (55 mg, 0.00785 mmol, 82% yield).

^{1}H NMR (500 MHz, DMSO-d_6) δ 8.24–8.01 (m, 12H, 12 x H-12), 4.94–3.25 (m, 192H, 12 x H-4, 12 x H-5, 12 x H-6, 12 x H-7, 12 x H-8, 12 x H-9, 12 x H-10, 12 x H-13, 12 x H-15), 2.68 (br, 12H, H-3a), 2.16–1.15 (m, 80H, 12 x H-3b, 12 x H-14, 12 x $NCOCH_3$, 12 x $OCOCH_3$).

^{13}C NMR (101 MHz, DMSO-d_6) δ 173.5 (C=O, $NCOCH_3$), 171.5 (C=O, C-1, 9-O-Ac), 163.57 (Cq, C-16), 145.7, 145.2 (C-11, C_{sp2}), 124.5 (C-12), 100.4 (C-2), 72.9 (C-6), 69.4 (C-18, C-8, C-7), 67.8 (C-4), 66.5 (C-9), 64.5 (C-15), 57.64 (C-10), 53.1 (C-5), 46.7 (C-17, C-13), 41.6 (C-3), 29.2 (C-14), 22.9 ($NCOCH_3$), 21.2 ($OCOCH_3$).

HRMS (ESI+-MS, m/z): calculated for $C_{306}H_{348}N_{48}O_{144}$ $[M+5H]^{5+}$ 1400.6350; found 1400.6368.

NMR Spectra

^{1}H NMR spectrum of **2** (CD_3OD, 500 MHz)

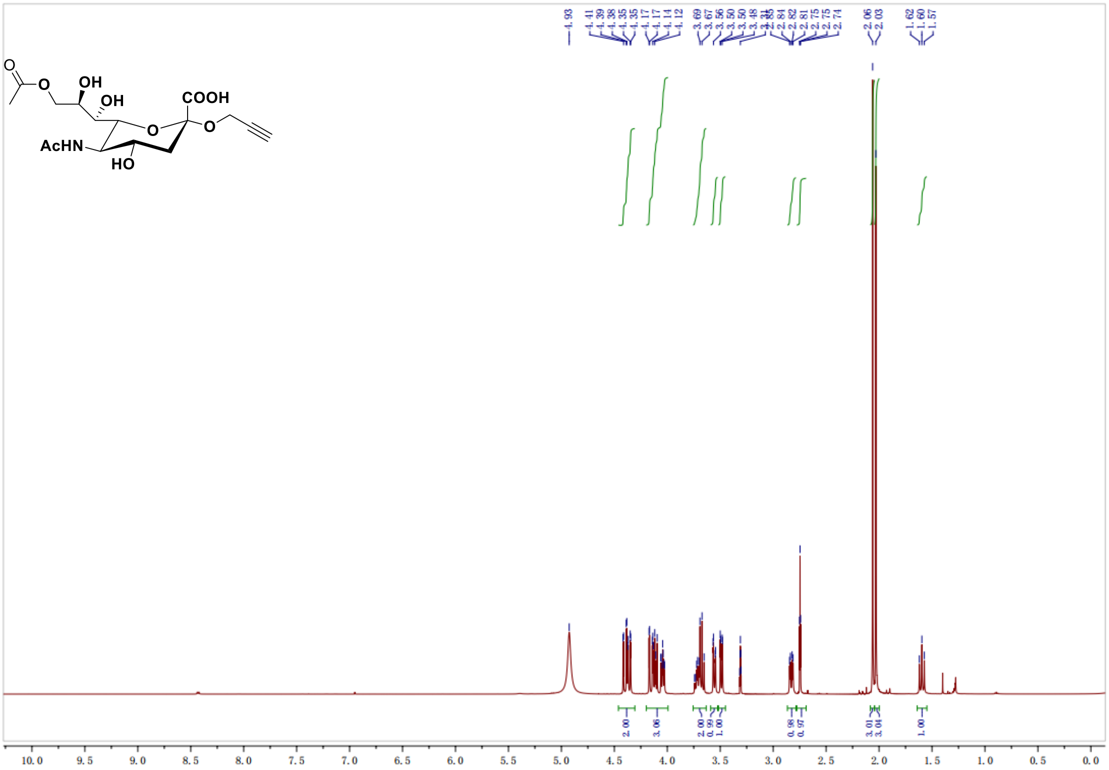

^{13}C NMR spectrum of **2** (CD_3OD, 126 MHz)

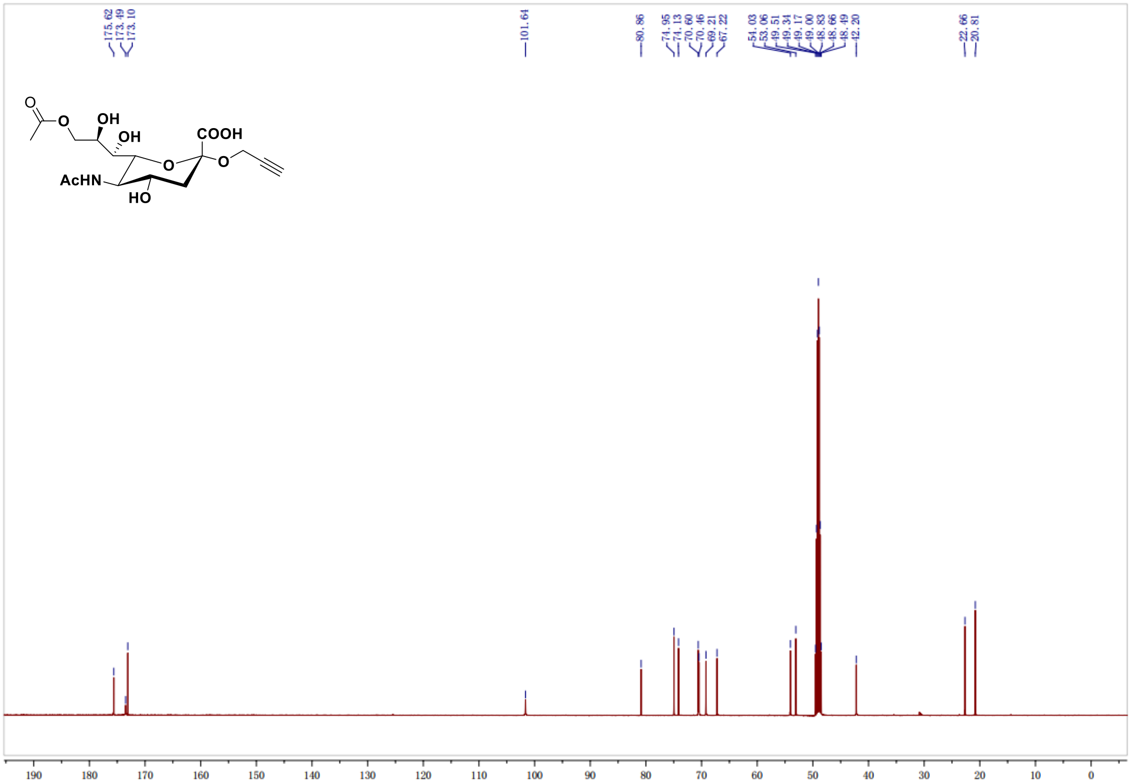

H-^{1}H Cosy spectrum of **2** (CD_3OD, 500 MHz)

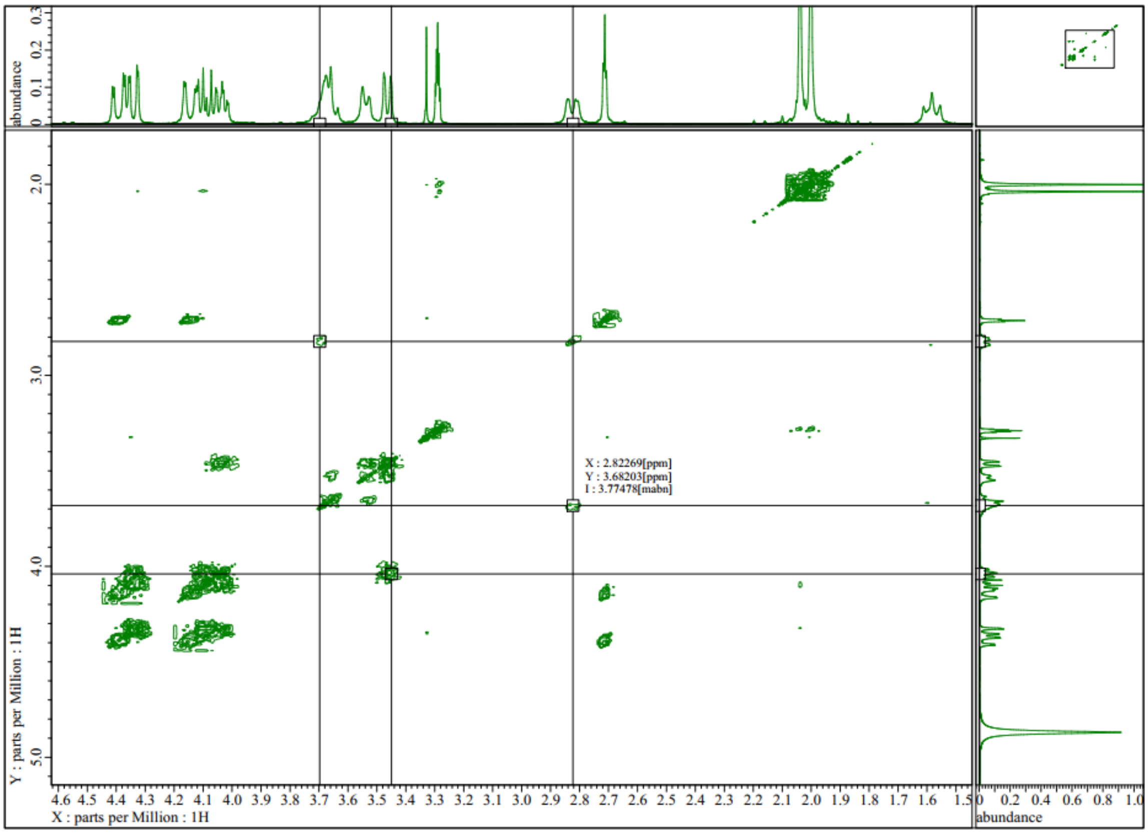

HMQC spectrum of **2** (CD_3OD, 500 MHz)

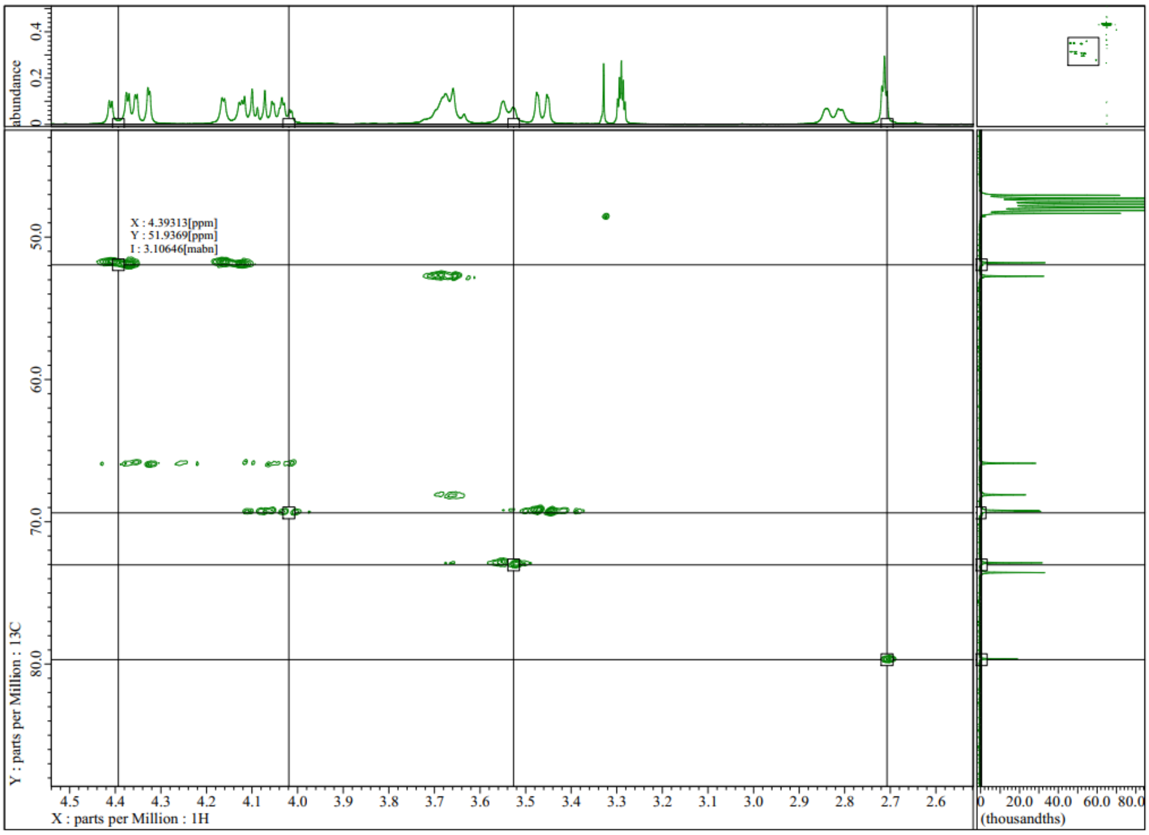

^{1}H NMR spectrum of **4** (DMSO-d$_6$, 500 MHz)

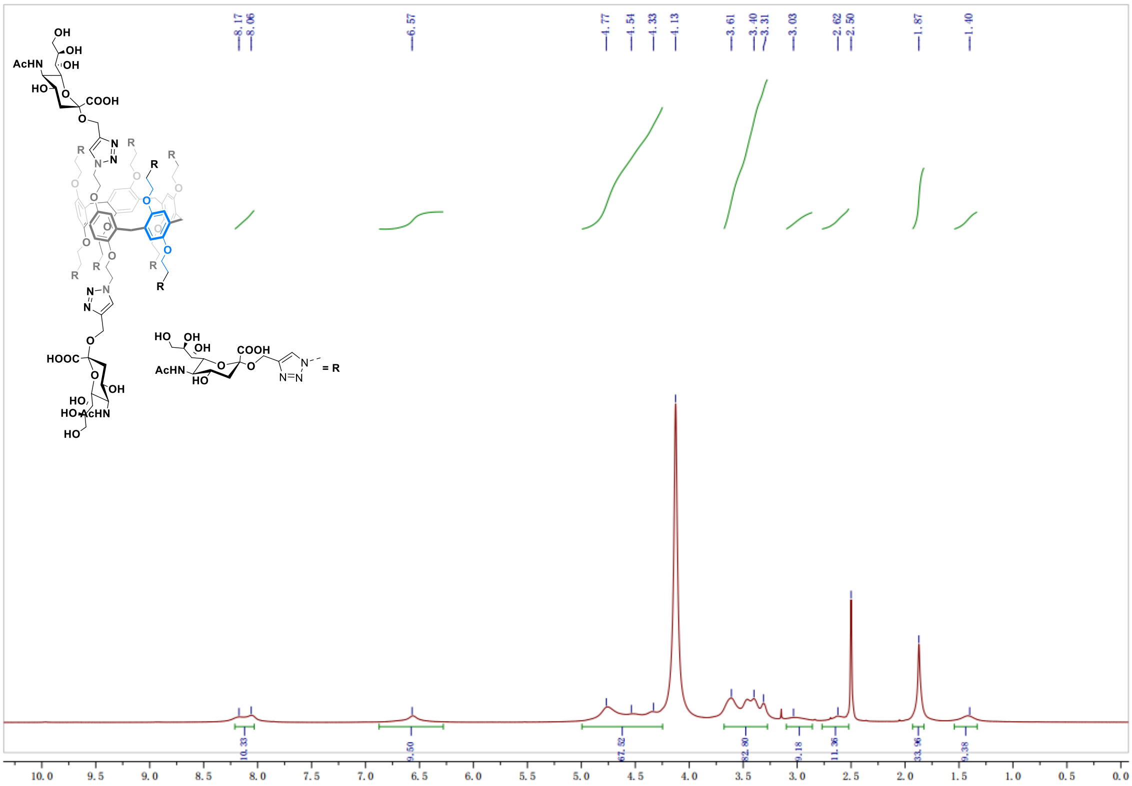

^{13}C NMR spectrum of **4** (DMSO-d$_6$, 126 MHz)

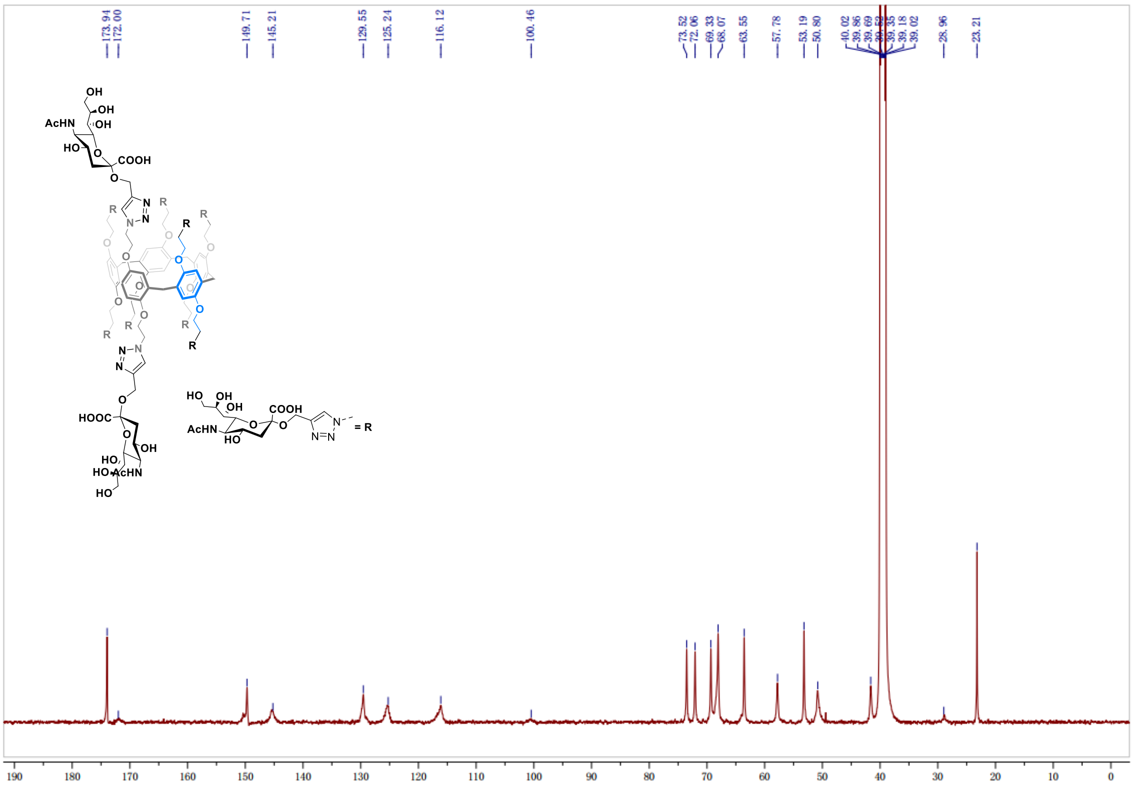

^{1}H NMR spectrum of **5** (CD_3OD, 500 MHz)

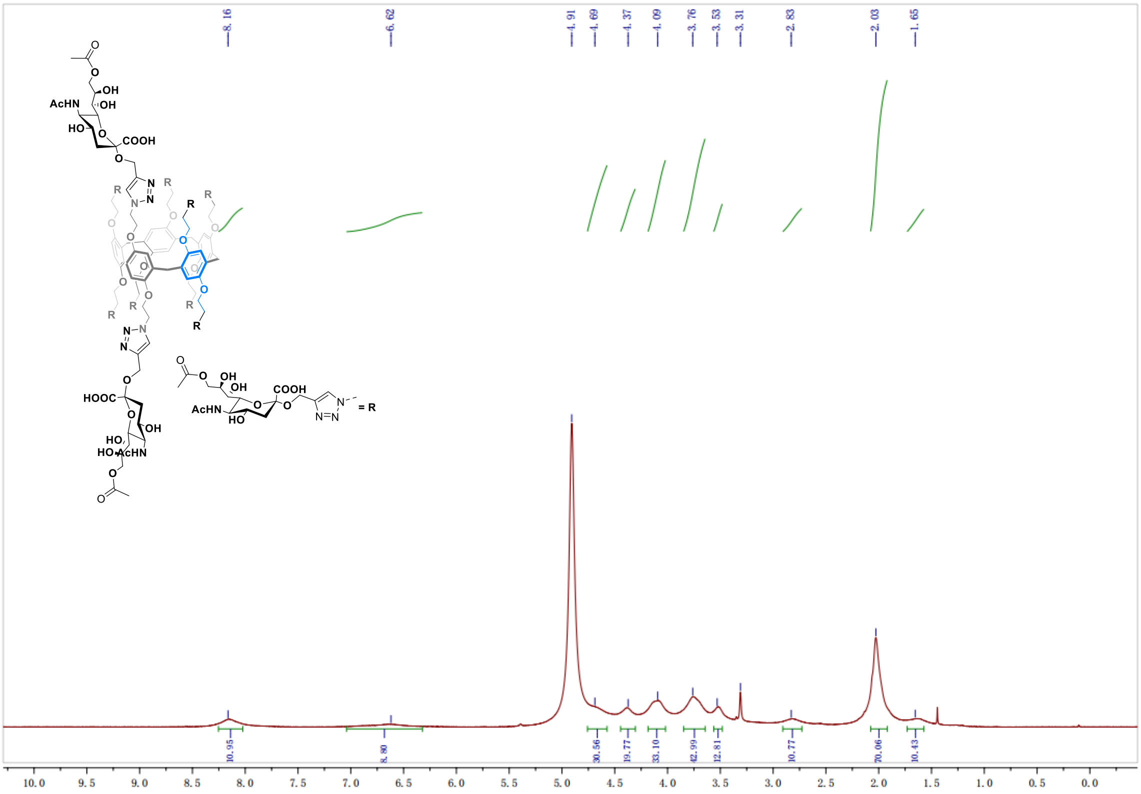

^{13}C NMR spectrum of **5** (CD_3OD, 126 MHz)

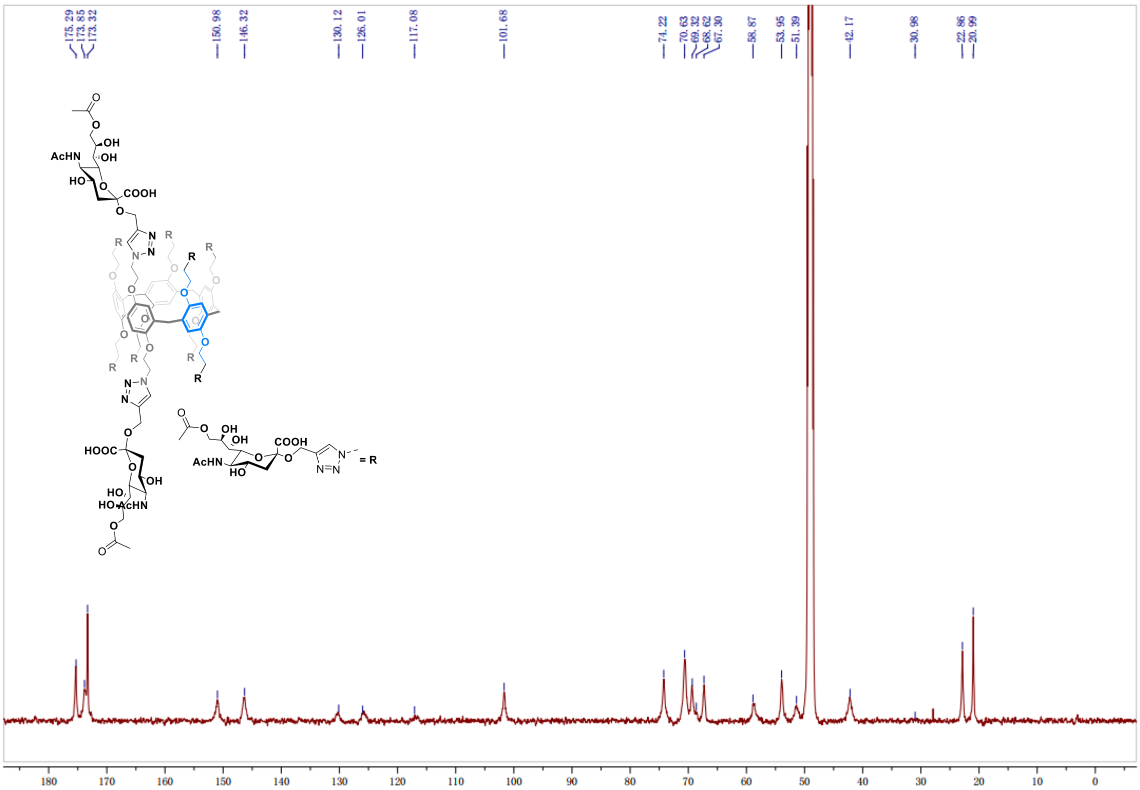

^{1}H NMR spectrum of **7** (CD_3OD, 500 MHz)

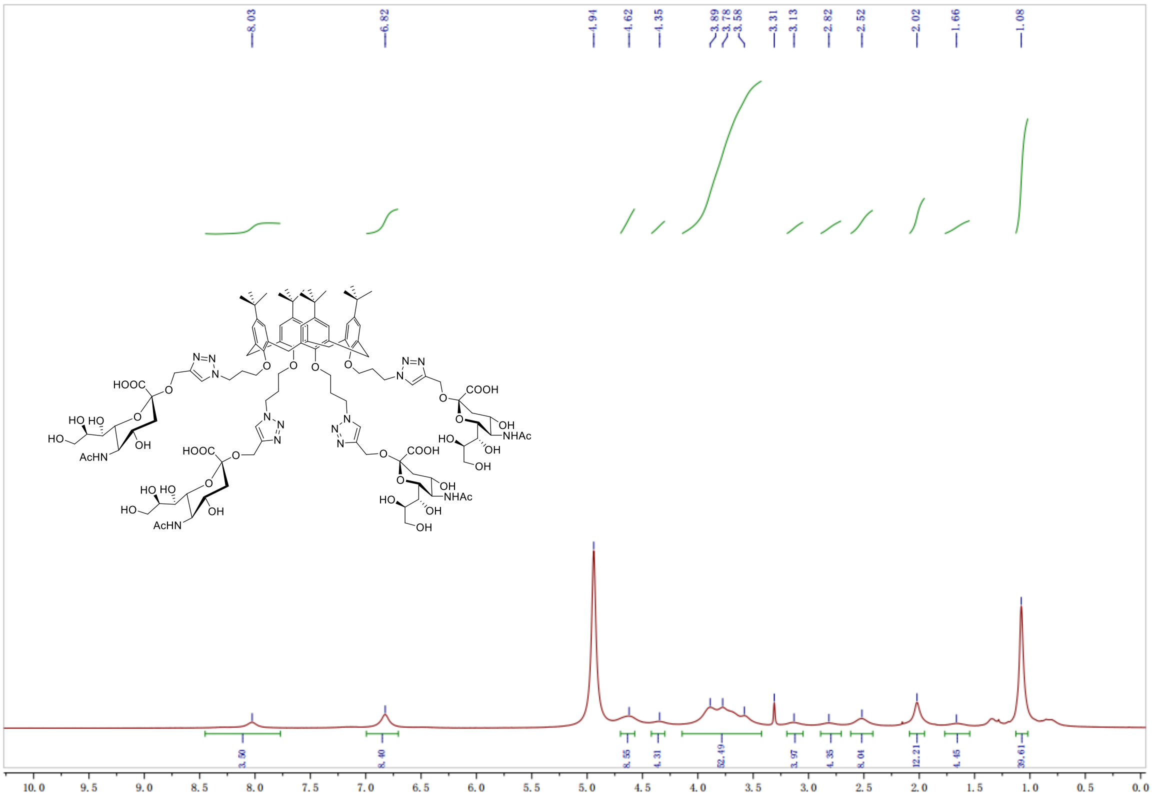

^{13}C NMR spectrum of **7** (CD_3OD, 126 MHz)

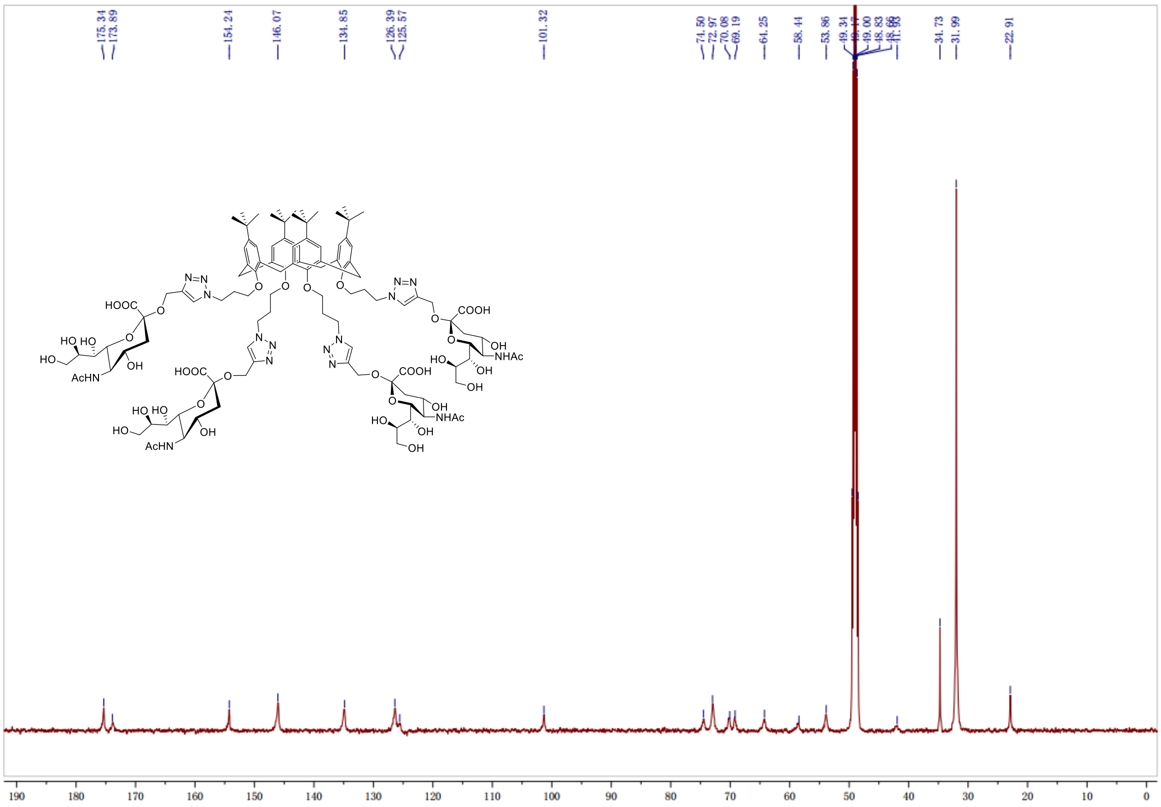

^{1}H NMR spectrum of **8** (CD_3OD, 500 MHz)

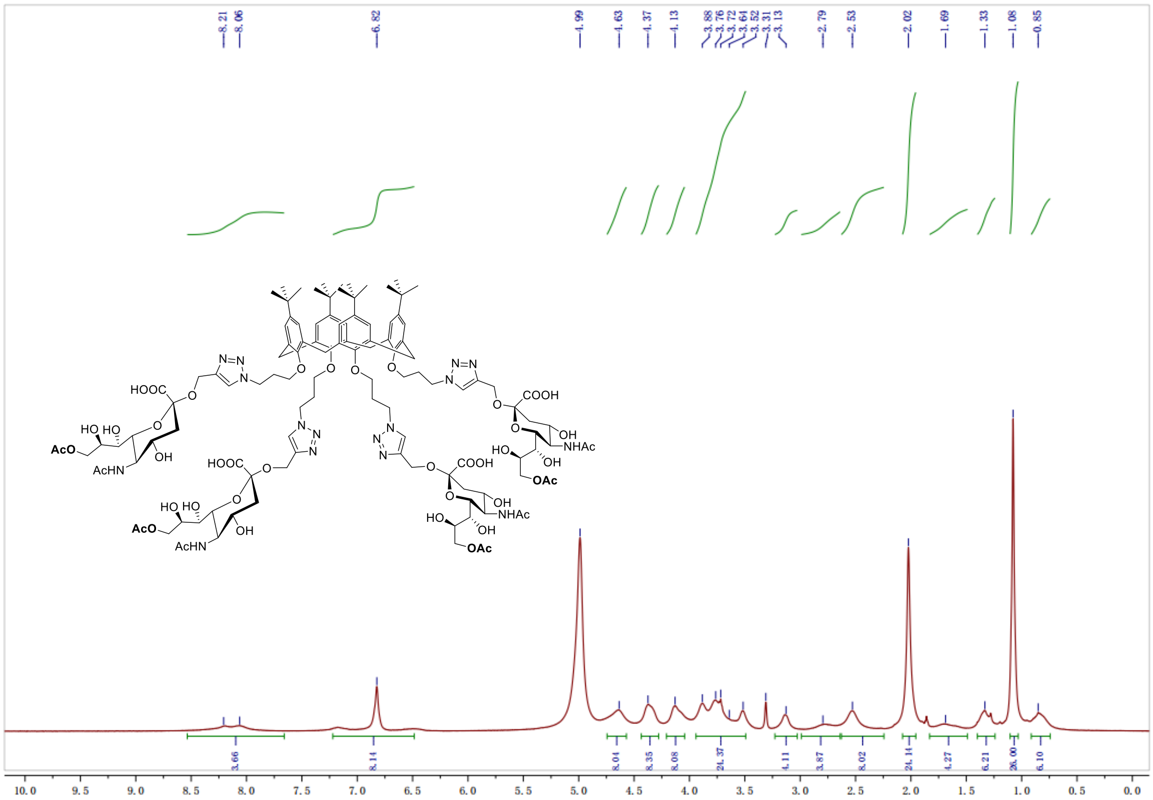

^{13}C NMR spectrum of **8** (CD_3OD, 126 MHz)

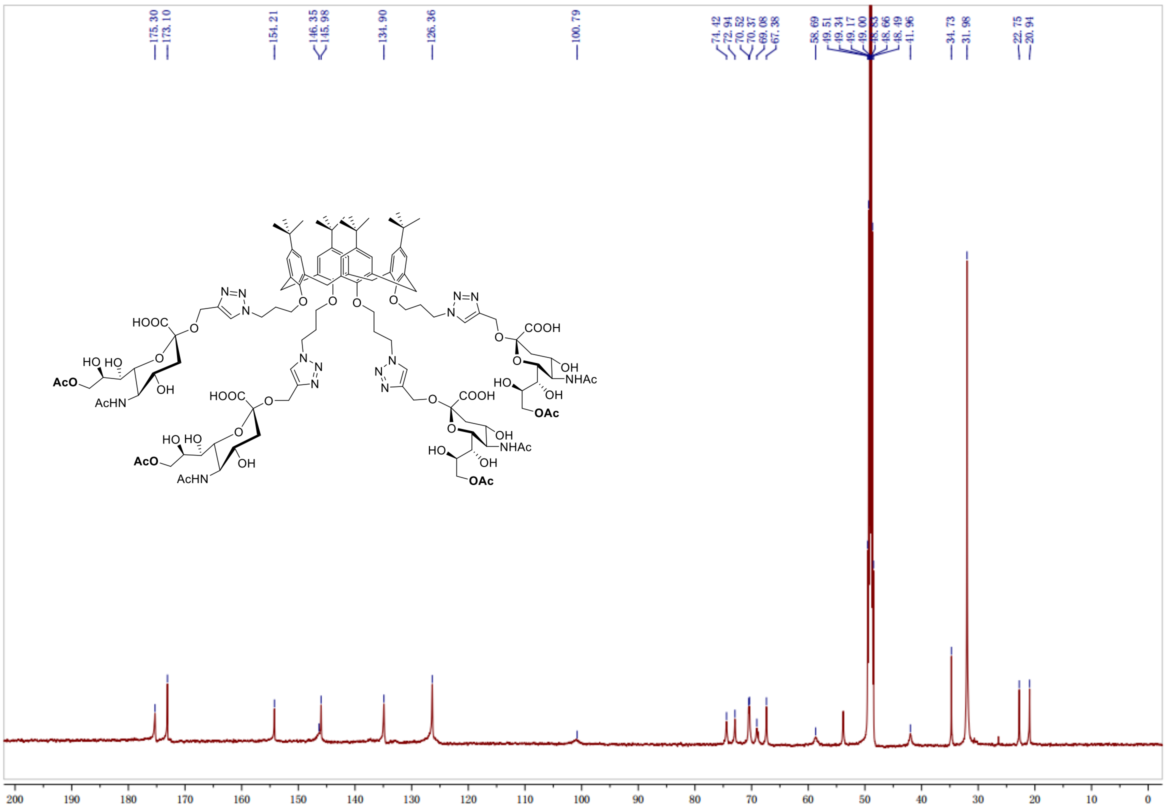

^{1}H NMR spectrum of **10** (D_2O/DMSO-d_6, 400 MHz)

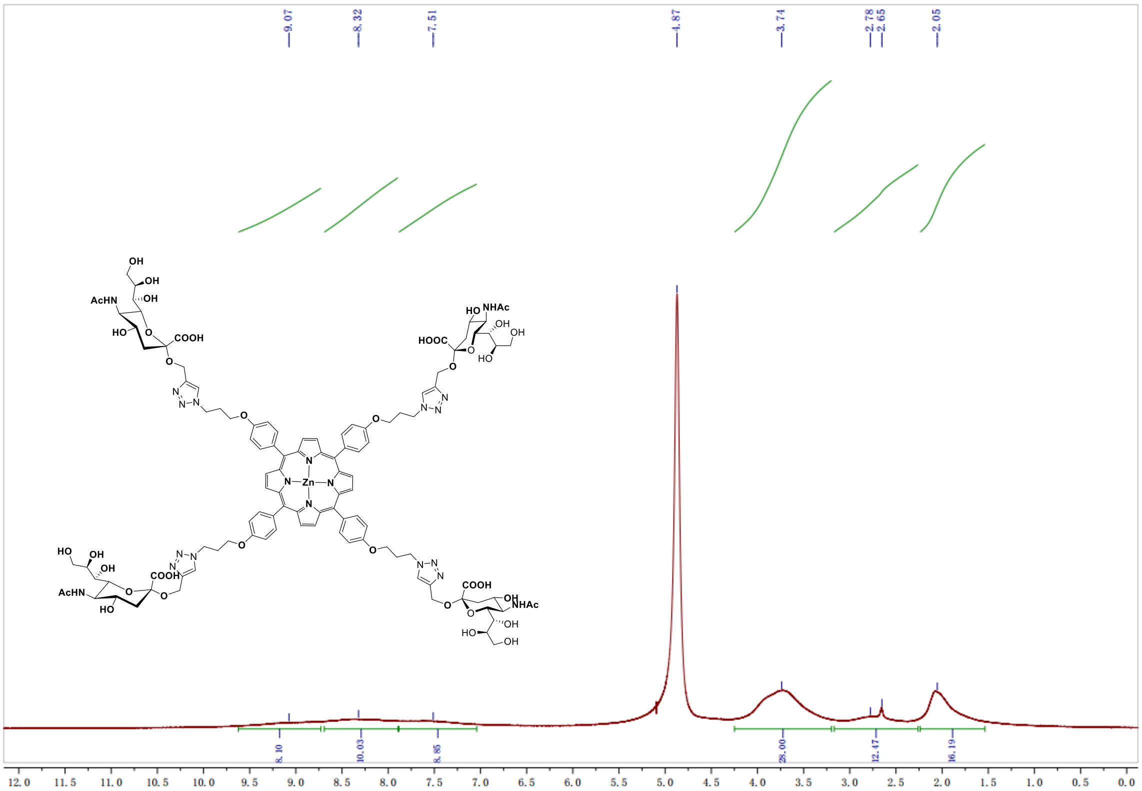

^{13}C NMR spectrum of **10** (D_2O/DMSO-d_6, 101 MHz)

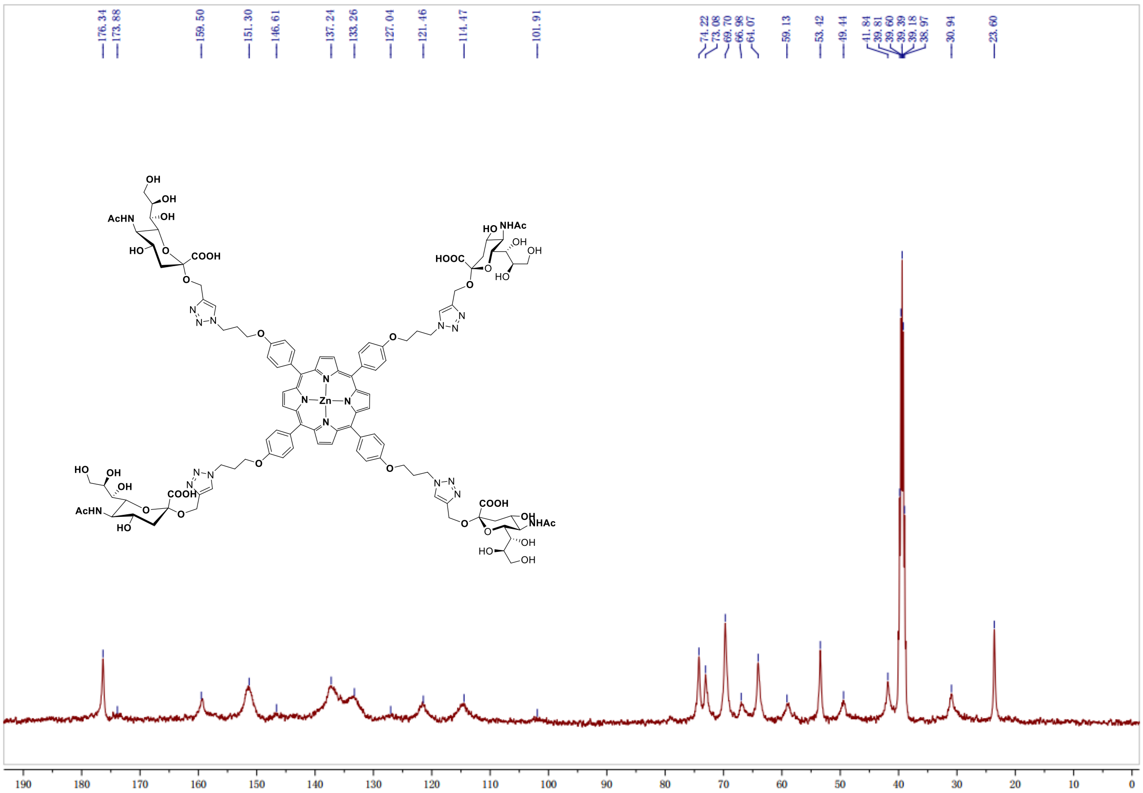

^{1}H NMR spectrum of **11** (DMSO-d$_6$, 500 MHz)

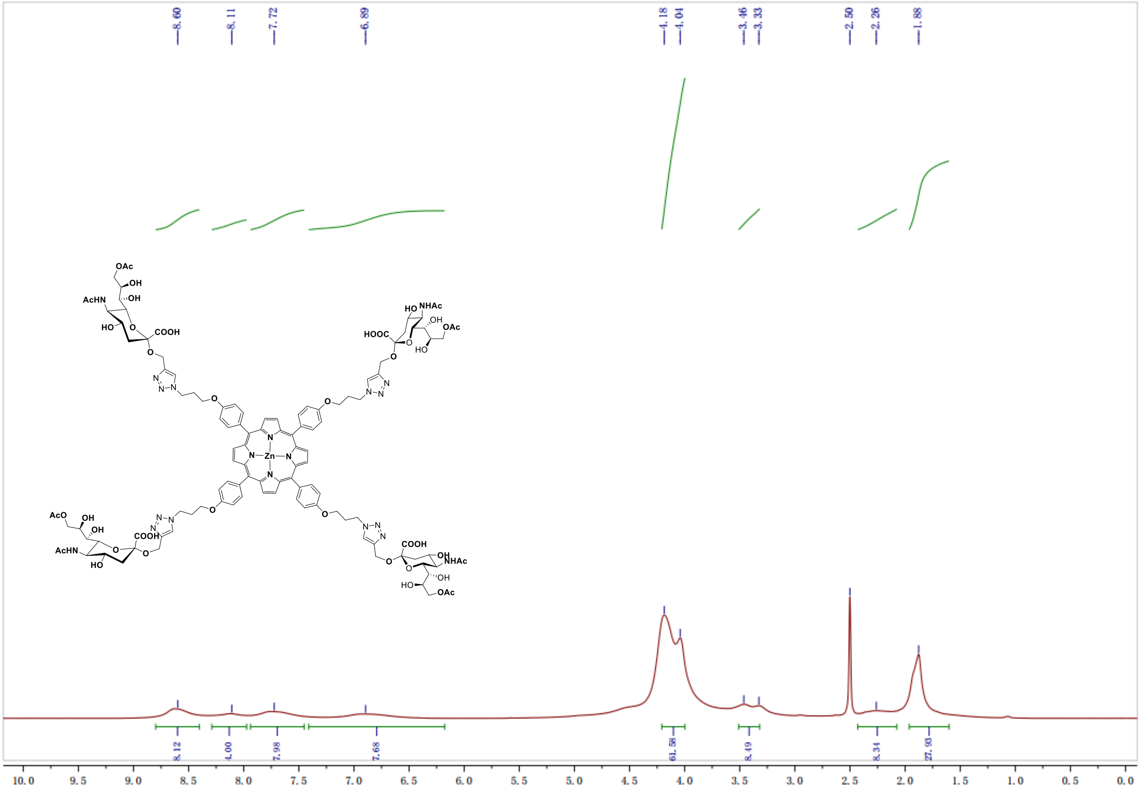

^{13}C NMR spectrum of **11** (DMSO-d$_6$, 126 MHz)

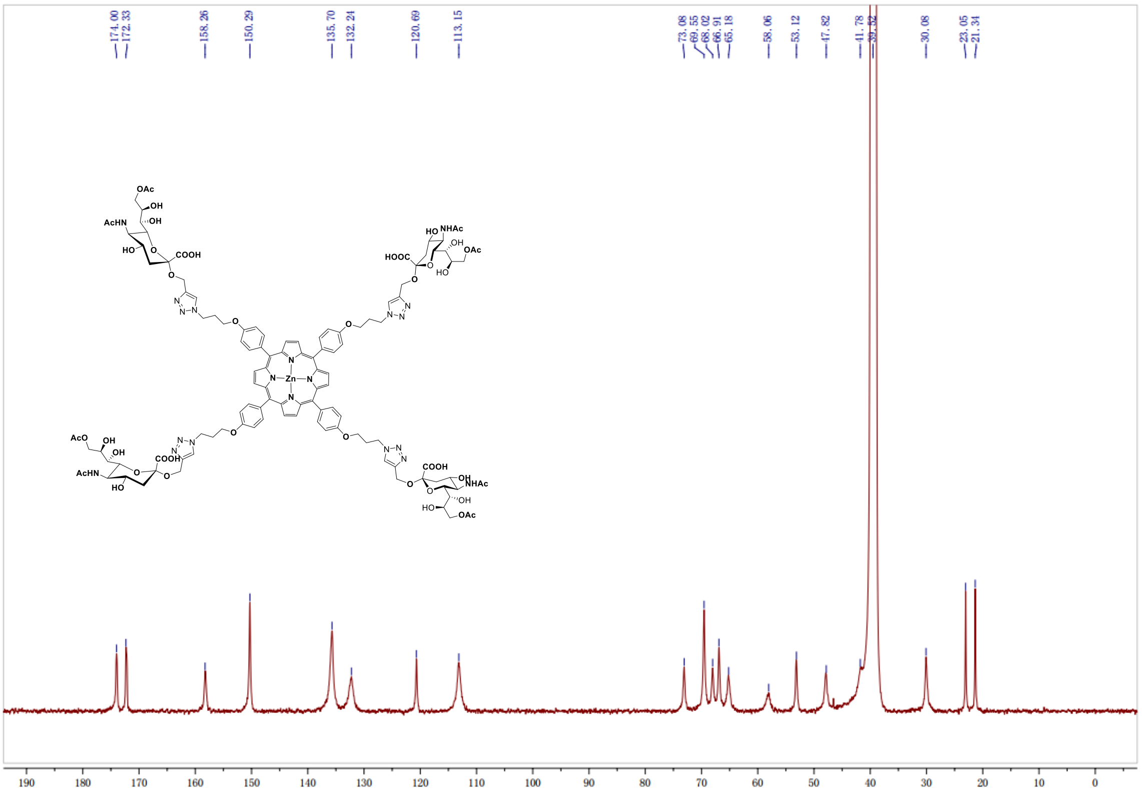

^{1}H NMR spectrum of **13** (DMSO-d$_6$, 400 MHz)

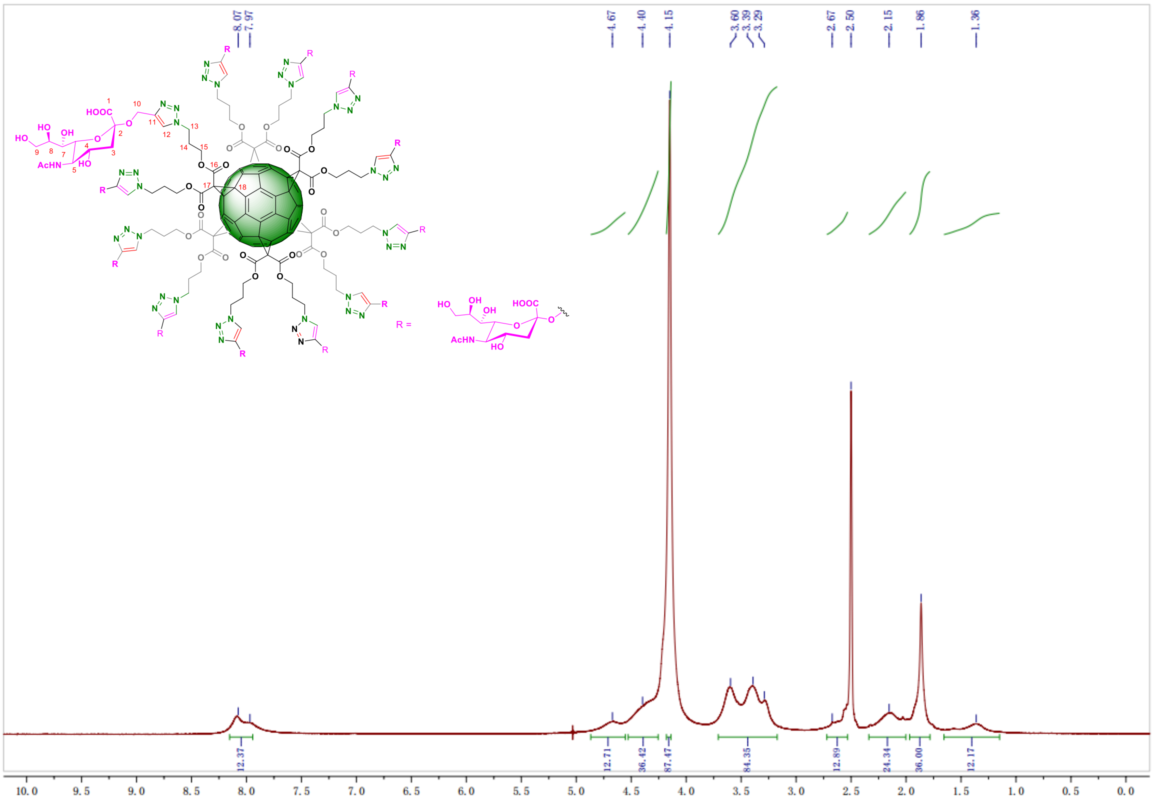

^{13}C NMR spectrum of **13** (DMSO-d$_6$, 101 MHz)

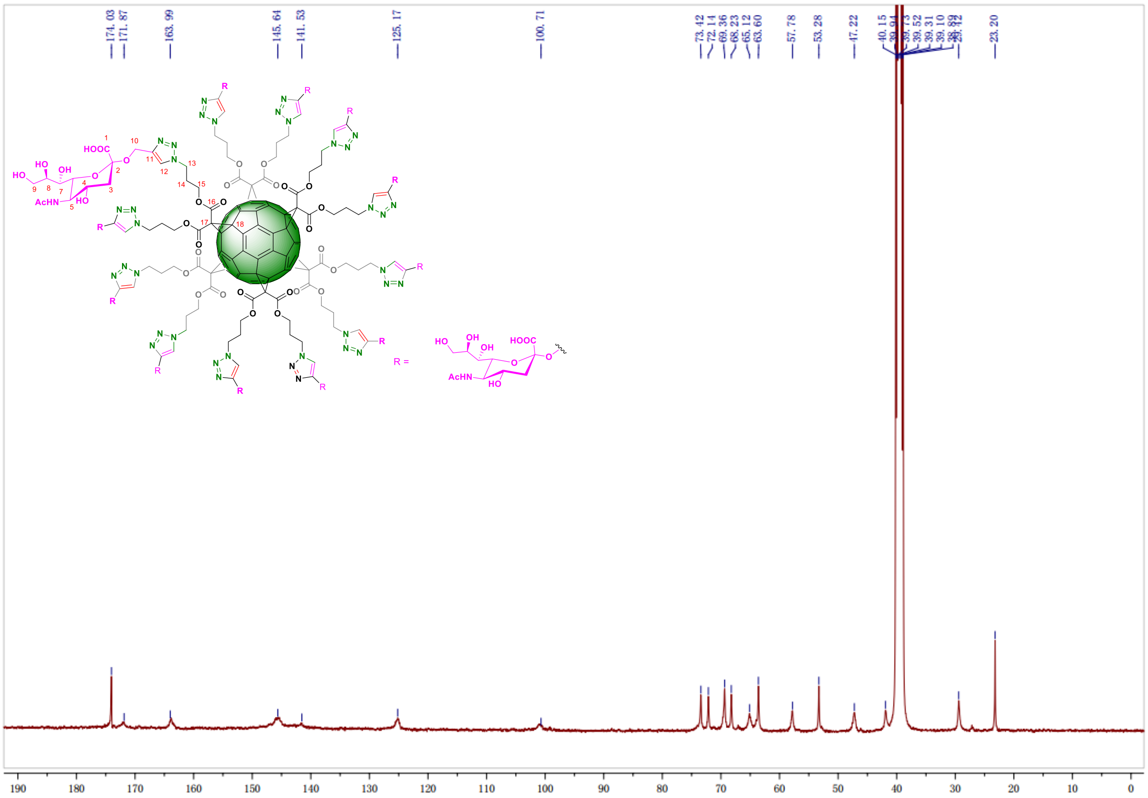

^{1}H NMR spectrum of **14** (DMSO-d$_6$, 500 MHz)

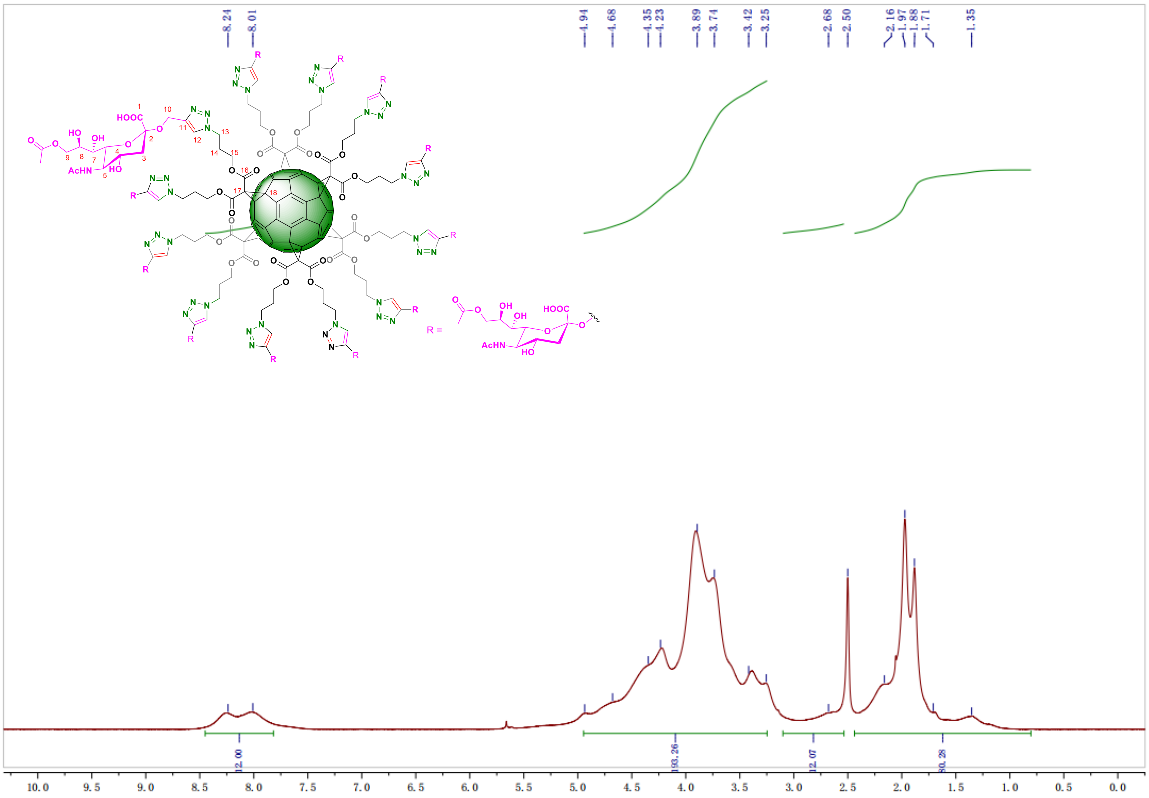

^{13}C NMR spectrum of **14** (DMSO-d$_6$, 400 MHz)

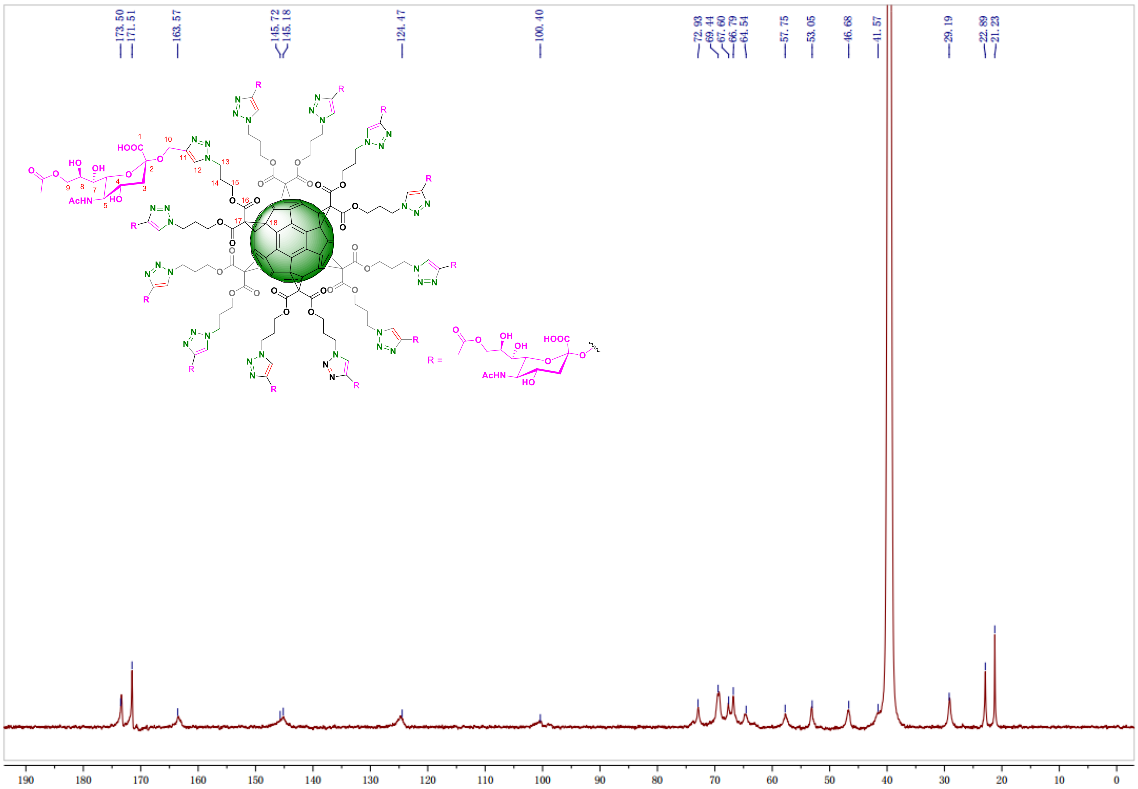

HMRS spectra

Compound **2**

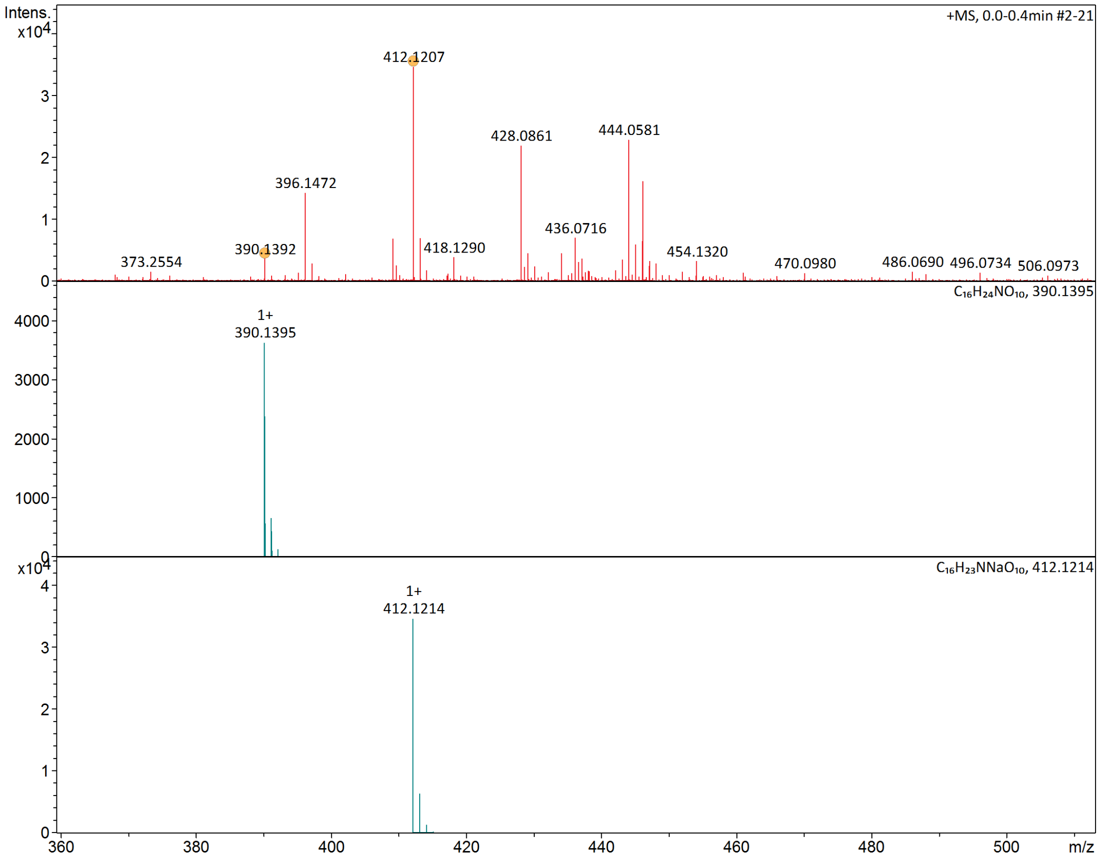

Compound **4**

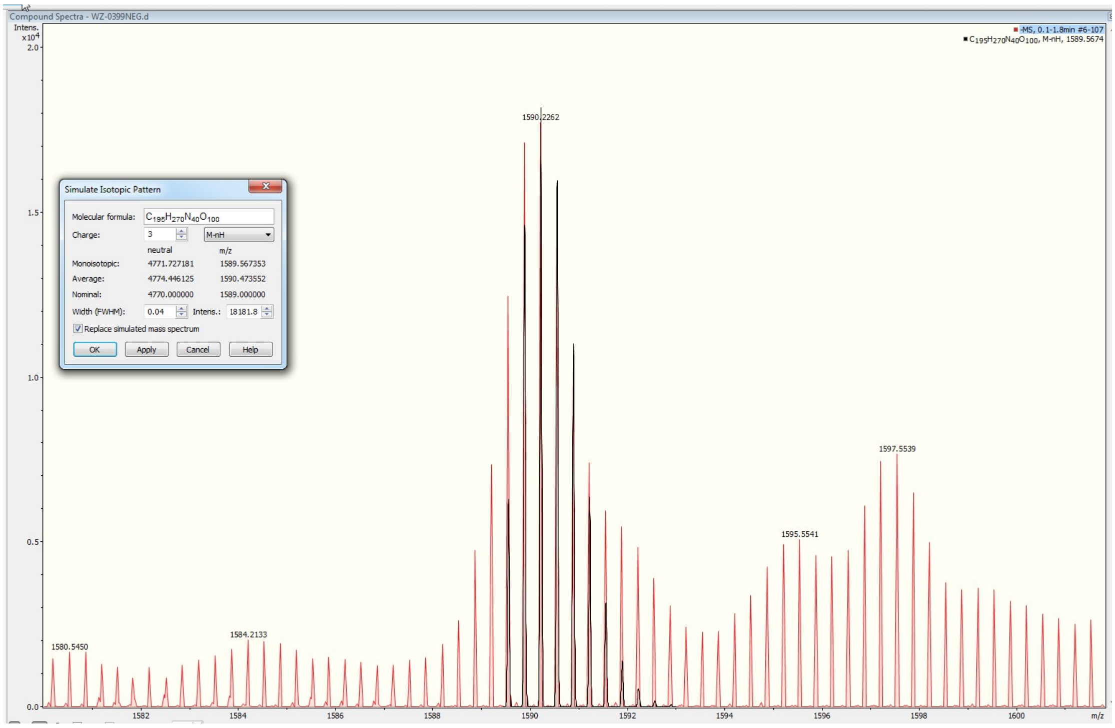

Predicted spectrum in black, measured spectrum in red.

Compound **5**

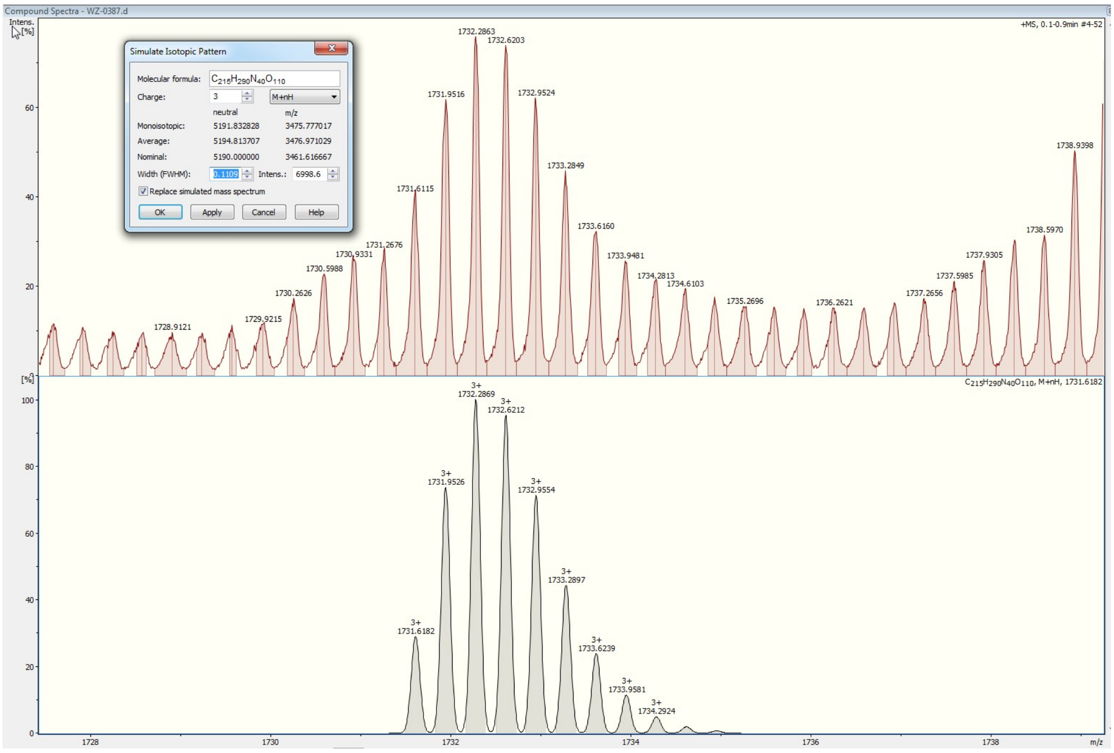

Predicted spectrum in black, measured spectrum in red.

Compound **7**

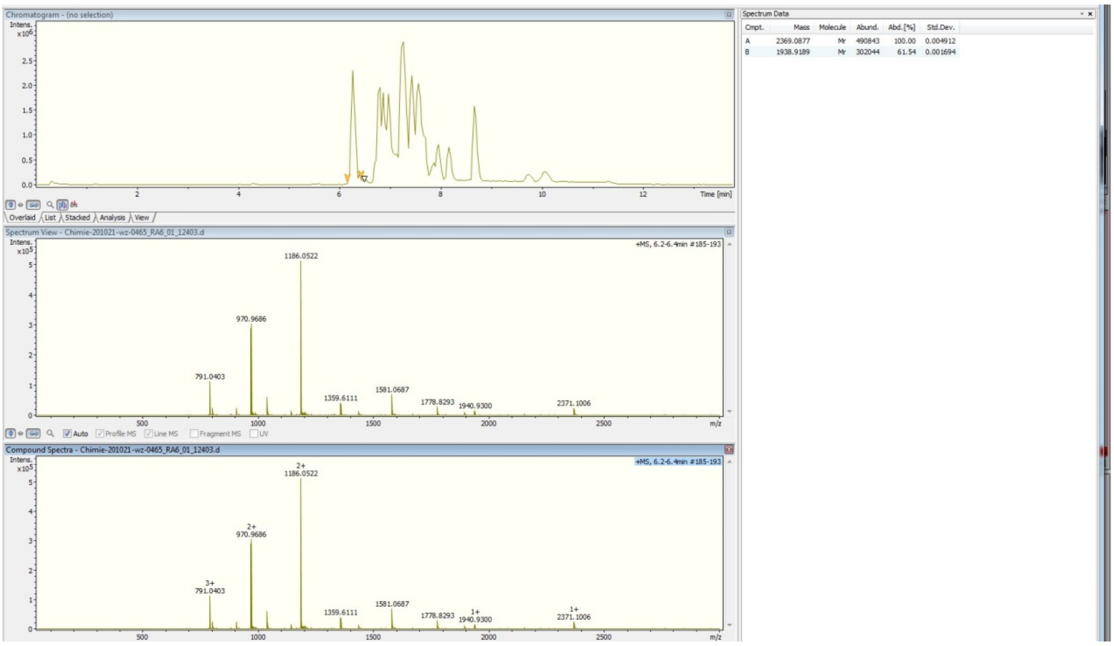

The Mass values presented in the Table were calculated after deconvolution.

Compound **8**

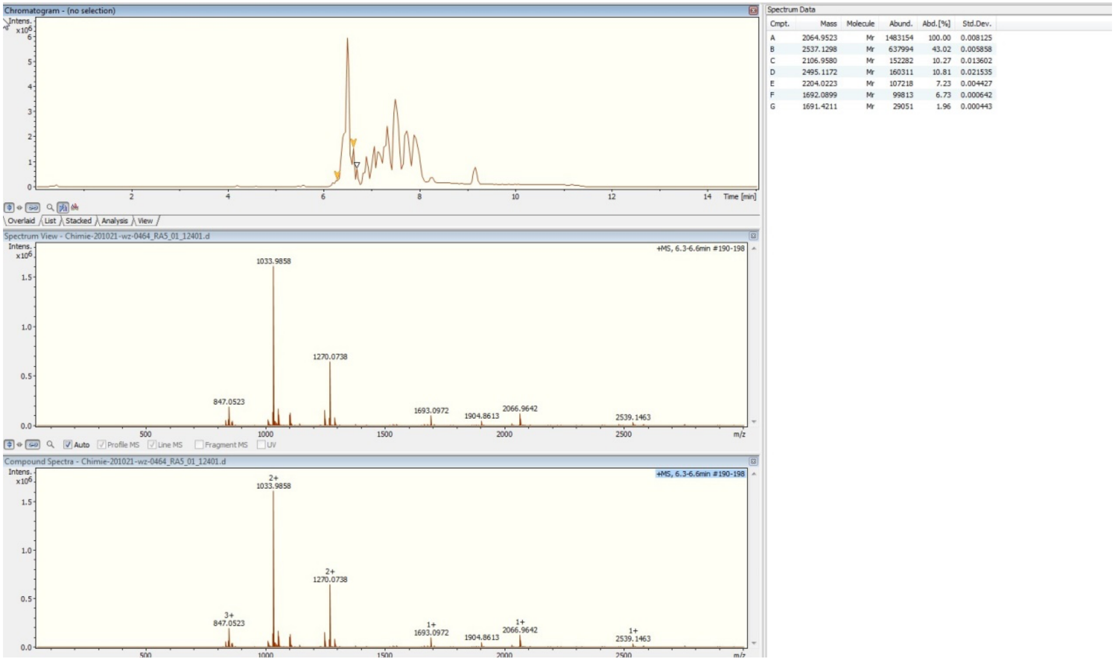

The Mass values presented in the Table were calculated after deconvolution.

Compound **10**

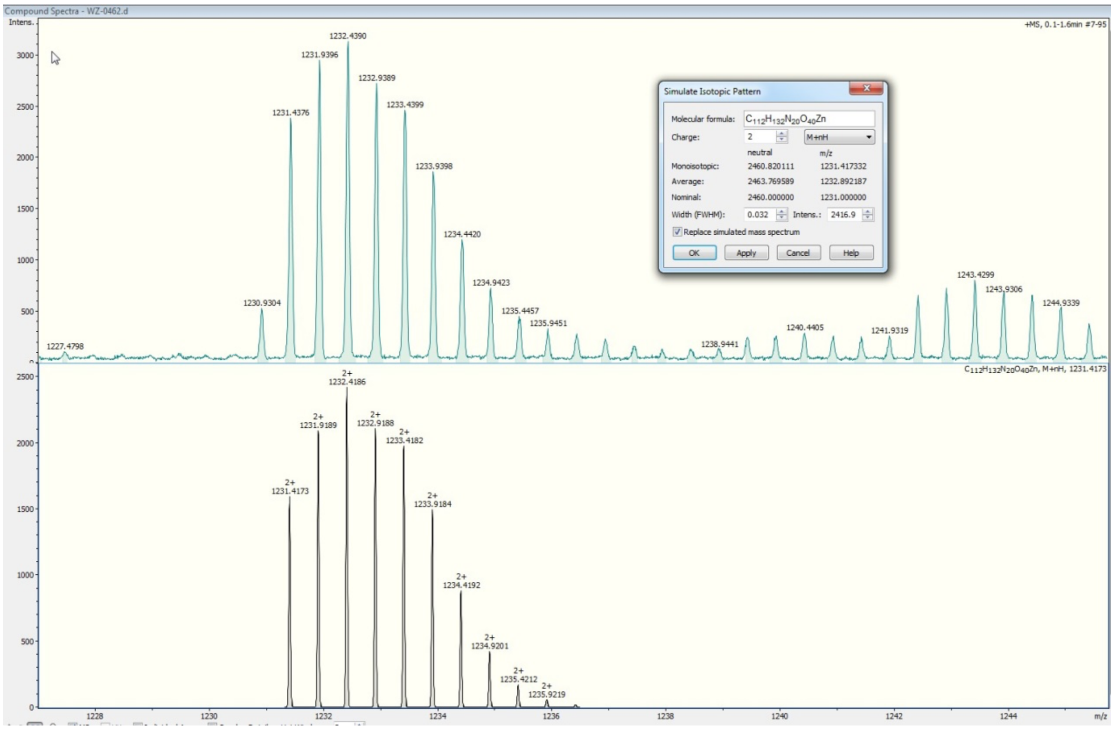

Predicted spectrum in black, measured spectrum in green.

Compound **11**

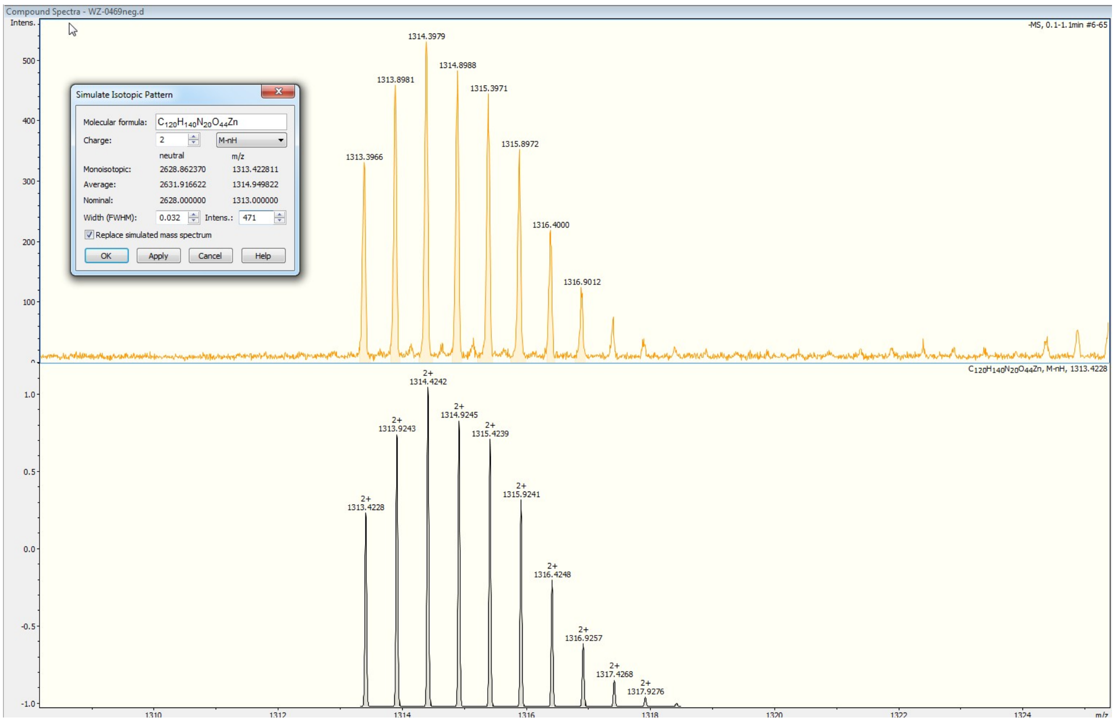

Predicted spectrum in black, measured spectrum in orange.

Compound **13**

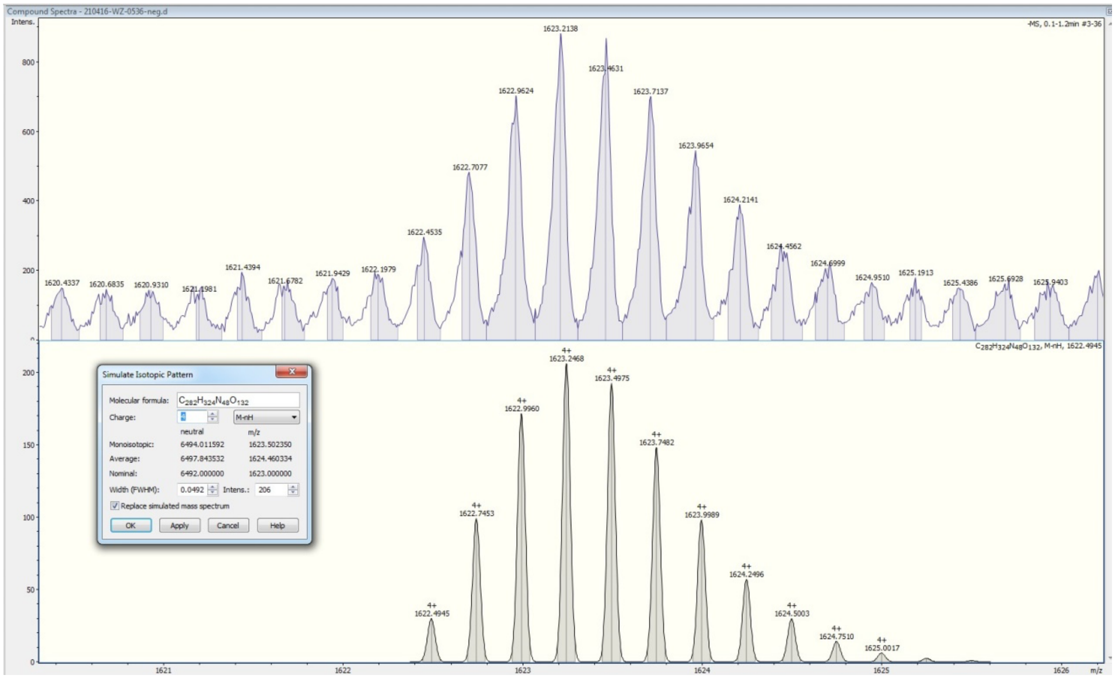

Predicted spectrum in black, measured spectrum in purple.

Compound **14**

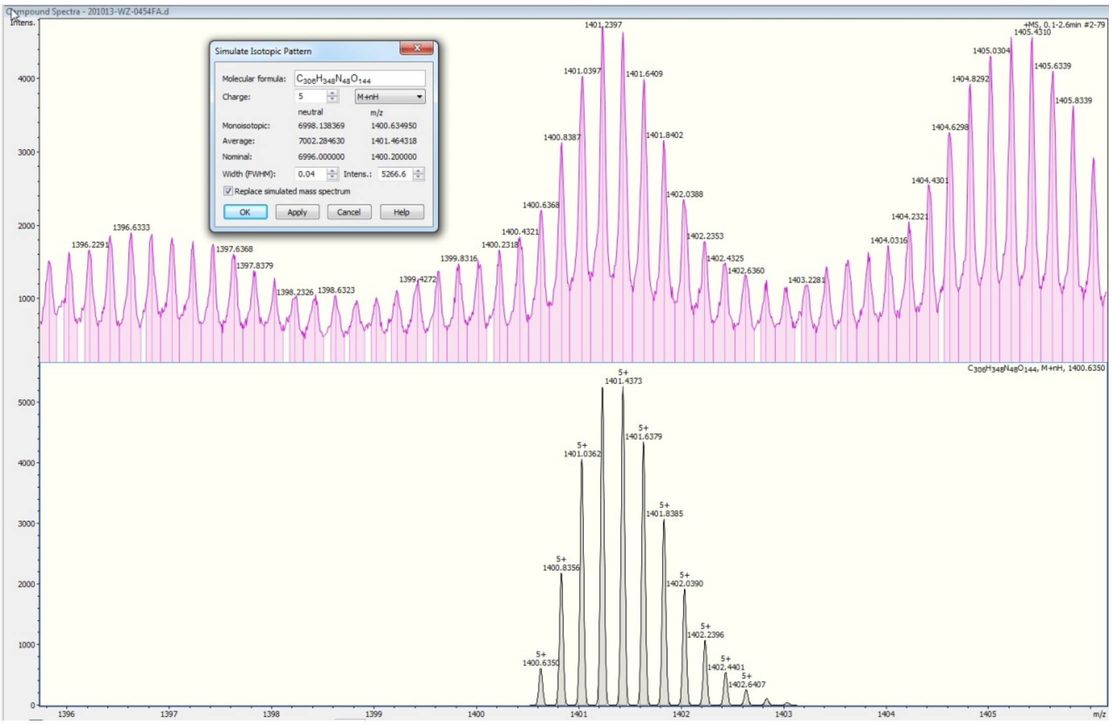

Predicted spectrum in black, measured spectrum in pink.

Supplementary Note 2: Synthesis and characterization of biotinylated sialic acids

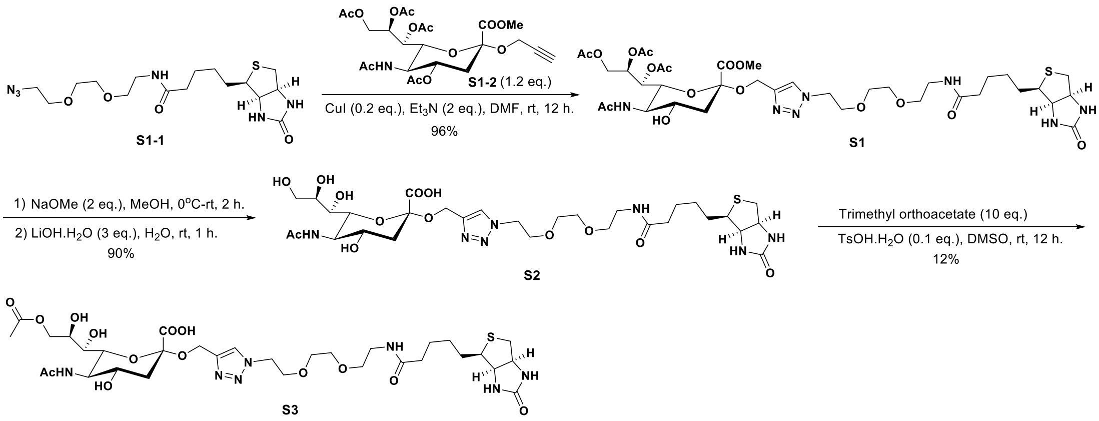

Scheme S3. Synthesis of compound **S2** and **S3**

Compound S1

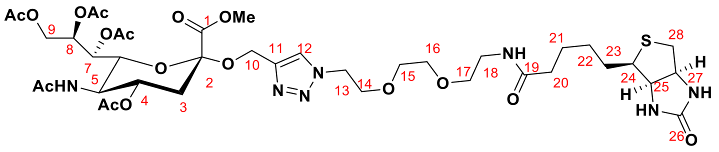

To a solution of **S1-1**[7] (401 mg, 1.0 mmol, 1 equiv.), **S1-2**[8] (636 mg, 1.2 mmol, 1.2 equiv.) and Et_3N (0.278 mL, 2.0 mmol, 2 equiv.) in dry DMF (20 mL) was added CuI (38 mg, 0.2 mmol, 0.2 equiv.) under argon atmosphere. The solution was stirred for 12 h at room temperature. After having evaporated the solvent under vacuum, EtOAc (30 mL) and saturated ammonium chloride (30 mL) were added, and the phases separated. The organic layer was washed with brine (30 mL), dried over $MgSO_4$, filtered and the filtrate was concentrated under reduced pressure. The residue was purified by column chromatography on silica gel using DCM/MeOH (20:1) as eluent to give a white solid (975 mg, 1.15 mmol, 96% yield).

^{1}H NMR (500 MHz, $CDCl_3$) 7.79 (s, 1H, H-12), 6.68 (br, 1H, NH), 6.43 (br, 1H, NH), 5.47 (d, *J* = 46.4 Hz, 1H, 10a), 5.45–5.42 (m, H-8), 5.35–5.32 (m, 1H, H-7), 4.92–4.87 (m, 2H, H-4, H-10b), 4.60–4.53 (m, 4H, H-13, H-17), 4.38–4.33 (m, 2H, H-9a, H-25), 4.16–4.05 (m, 3H, H-5, H-6, H-9b), 3.91 (t, *J* = 5.1 Hz, 2H, H-14), 3.80 (s, 3H, $COCH_3$), 3.61 (dd, *J* = 6.0, 2.7 Hz, 2H, H-15), 3.56 (dd, *J* = 5.9, 2.7 Hz, 2H, H-15), 3.50 (t, *J* = 5.2 Hz, 2H, H-17), 3.41–3.40 (m, 2H, H-18), 3.17 (dd, *J* = 11.8, 7.3 Hz, 1H, H-24), 2.95–2.88 (m, 1H, H-28a), 2.77 (d, *J* = 12.9 Hz, 1H, H-3a), 2.62 (dd, *J* = 12.8, 4.6 Hz, 1H, H-28b), 2.24 (t, *J* = 7.5 Hz, 2H, H-20), 2.16 (s, 3H, $OCOCH_3$), 2.15 (s, 3H,

$OCOCH_3$), 2.03 (s, 3H, $OCOCH_3$), 2.02 (s, 3H, $OCOCH_3$), 1.97 (t, *J* = 12.5 Hz, 1H, H-3b), 1.88 (s, 3H, $NCOCH_3$), 1.77–1.63 (m, 4H, H-21, H-23), 1.48–1.42 (m, 2H, H-22).

^{13}C NMR (126 MHz, $CDCl_3$) δ 173.5 (C-19), 171.0, 170.80, 170.5, 170.3, 170.2 (C=O, $NCOCH_3$, OAc), 168.2 (C-1), 164.0 (Cq, C-26), 143.8 (C-11), 124.4 (C-12), 98.6 (C-2), 72.8 (C-6), 70.4 (C-16), 70.1 (C-17), 69.9 (C-15), 69.4 (C-14), 69.2 (C-4), 68.5 (C-8), 67.5 (C-7), 62.6 (C-9), 61.9 (C-25), 60.3 (C-27), 58.4 (C-10), 55.7 (C-24), 53.0 (OCH_3), 50.2 (C-13), 49.2 (C-5), 40.5 (C-28), 39.1 (C-18), 37.9 (C-3), 35.9 (C-20), 28.3 (C-22), 28.1 (C-23), 25.6 (C-21), 23.2 ($NCOCH_3$), 21.2, 21.0, 2 x 20.9 ($OCOCH_3$).

HRMS (TOF-MS-ESI$^+$, m/z): calculated for $C_{39}H_{60}N_7O_{17}S$ $[M+H]^+$ 930.3761; found 930.3750.

Compound S2

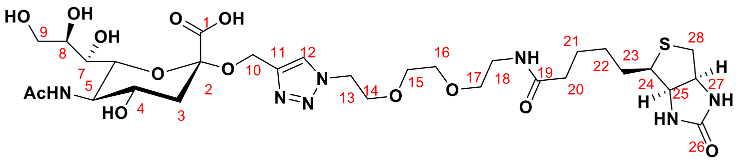

A solution of **S1** (345 mg, 0.371 mmol, 1 equiv.) and NaOMe (40 mg, 0.742 mmol, 2 equiv.) in dry MeOH (20 mL) was stirred at 0ºC for 30 min, then warmed up to room temperature and stirred for another 1.5 h. Afterwards, Amberlyst®15 ion-exchange resin was added to neutralize the base. The resin was filtered and washed with water (2 x 5 mL). The filtrate was evaporated under reduced pressure to afford a white solid. Without further purification, the white solid was dissolved in water (15 mL) and $LiOH.H_2O$ (35 mg, 0.831 mmol, 3 equiv.) was added. The solution was stirred at room temperature for 1 h before adding Amberlyst®15 ion-exchange. Afterwards, the reaction mixture was filtered, the resin was washed with water (2 x 5 mL) and the filtrate was lyophilized to obtain white solid (186 mg, 0.249 mmol, 90% yield).

^1H NMR (500 MHz, D_2O) δ 8.06 (s, 1H, H-12), 4.92–4.89 (m, 1H, H-10a), 4.68–4.66 (m, 1H, H-10b), 4.62–4.60 (m, 2H, H-13), 4.57–4.53 (m, 1H, H-27), 4.38–4.34 (m, 1H, H-25), 3.95–3.93 (m, 2H, H-14), 3.88–3.79 (m, 4H, H-5, H-8, H-6, H-9a), 3.76–3.71 (m, 1H, H-4), 3.65–3.55 (m, 6H, H-15, H-16, H-9b, H-7), 3.52–3.49 (m, 2H, H-17), 3.32–3.30 (m, 2H, H-18), 3.27–3.24 (m, 1H, H-24), 2.95–2.90 (m, 1H, H-28a), 2.74–2.68 (m, 2H, H-3a, H-28b), 2.21 (dd, *J* = 13.2, 6.5 Hz, 2H, H-20), 2.01–2.00 (m, 3H, NAc), 1.74 (t, *J* = 12.2 Hz, 1H, H-3b), 1.61–1.48 (m, 4H, H-21, H-23), 1.36–1.33 (m, 2H, H-22).

^{13}C NMR (126 MHz, D_2O) δ 176.8 (C=O, $NCOCH_3$), 175.0 (C-1), 171.8 (C=O, C-19), 165.3 (C=O, C-26), 143.6 (C-11), 125.7 (C-12), 99.5 (C-2), 72.9 (C-6), 71.1 (C-8), 69.7 (C-16), 69.3 (C-17), 68.9 (C-15), 68.7 (C-14), 68.3 (C-7), 67.7 (C-4), 62.9 (C-9), 62.1 (C-25), 60.3 (C-27), 57.2 (C-10), 55.4 (C-24), 51.8 (C-5), 50.1 (C-13), 2 x 39.7 (C-28, C-3), 38.9 (C-18), 35.5 (C-20), 27.9 (C-22), 27.7 (C-23), 25.2 (C-21), 22.1 ($NCOCH_3$).

HRMS (TOF-MS-ESI$^+$, m/z): calculated for $C_{30}H_{50}N_7O_{13}S$ $[M+H]^+$ 748.3182; found 748.3173.

Compound S3

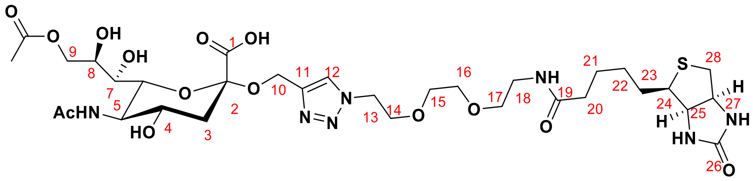

The title compound was prepared following a literature procedure.[5] To a solution of compound **S2** (200 mg, 0.267 mmol, 1 equiv.) and trimethyl orthoacetate (0.34 mL, 2.67 mmol, 10 equiv.) in dry DMSO (1.2 mL) was added *p*-toluenesulfonic acid monohydrate (5.0 mg, 0.027 mmol, 0.1 equiv.). The solution was stirred at room temperature for 12 h. Then, DCM (50 mL) was added to precipitate the crude product. The crude was purified by C_{18} silica gel flash chromatography using H_2O/MeOH (0-1/3, gradient) as eluent. The fractions containing **5** were combinded and concentrated under reduced pressure. The concentrated solution was lyophilized to afford the desired compound as a white solid (25 mg, 0.0316 mmol, 12%).

^1H NMR (500 MHz, CD_3OD) δ 8.02 (s, 1H, H-12), 4.94 (d, *J* = 12.1 Hz, 1H, H-10a), 4.67 (d, *J* = 12.1 Hz, 1H, H-10b), 4.55–4.55 (m, 2H, H-13), 4.49 (dd, *J* = 7.8, 4.7 Hz, 1H, H-27), 4.38 (dd, *J* = 11.2, 1.8 Hz, 1H, H-25), 4.31 (dd, *J* = 7.9, 4.5 Hz, 1H, H-9a), 4.14–4.06 (m, 2H, H-8, H-9b), 3.90 (t, *J* = 5.1 Hz, 2H, H-14), 3.74–3.71 (m, 2H, H-5, H-4), 3.64–3.60 (m, 3H, H-6, H-16), 3.58–3.56 (m, 2H, H-17), 3.51–3.48 (m, 3H, H-7, H-15), 3.35–3.33 (m, 2H, H-18), 3.20–3.17 (m, 1H, H-24), 2.94–2.84 (m, 2H, H-3a, H-28a), 2.69 (d, *J* = 12.7 Hz, 1H, H-28b), 2.21 (t, *J* = 7.4 Hz, 2H, H-20), 2.05 (s, $OCOCH_3$), 2.02 (s, $NCOCH_3$), 1.74–1.71 (m, 1H, H-3b), 1.66–1.5 (m, 4H, H-21, H-23), 1.45–1.41 (m, 2H, H-22).

^{13}C NMR (126 MHz, CD_3OD) δ 176.2 (C=O, $NCOCH_3$), 175.5 (C-1), 174.0 (C=O, 9-O-Ac), 173.1 (C=O, C-19), 166.1 (C=O, C-26), 146.5 (C-11), 125.9 (C-12), 101.9 (C-2), 74.2 (C-6), 71.4 (C-16), 71.2 (C-17), 70.7 (C-8), 2 x 70.6 (C-7, C-15), 70.3 (C-14), 69.6 (C-4), 67.3 (C-9), 63.3 (C-25), 61.6

(C-27), 58.9 (C-10), 57.0 (C-24), 54.1 (C-5), 51.3 (C-13), 42.6 (C-3), 41.1 (C-28), 40.3 (C-18), 36.7 (C-20), 29.7 (C-22), 29.5 (C-23), 26.8 (C-21), 22.6 ($NCOCH_3$), 20.8 ($OCOCH_3$).

HRMS (TOF-MS-ESI$^+$, m/z): calculated for $C_{32}H_{52}N_7O_{14}S$ $[M+H]^+$ 790.3287; found 790.3287.

Characterization

S1 ($CDCl_3$, 500 MHz)

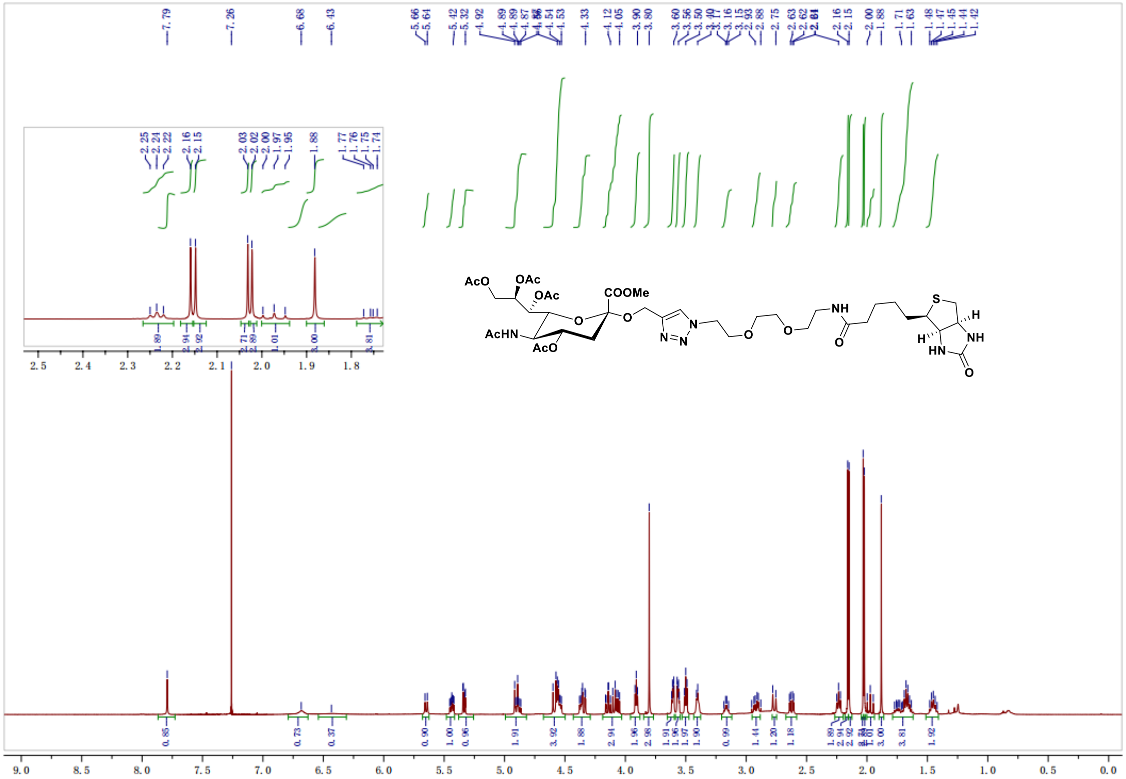

^{13}C NMR spectrum of **S1** ($CDCl_3$, 126 MHz)

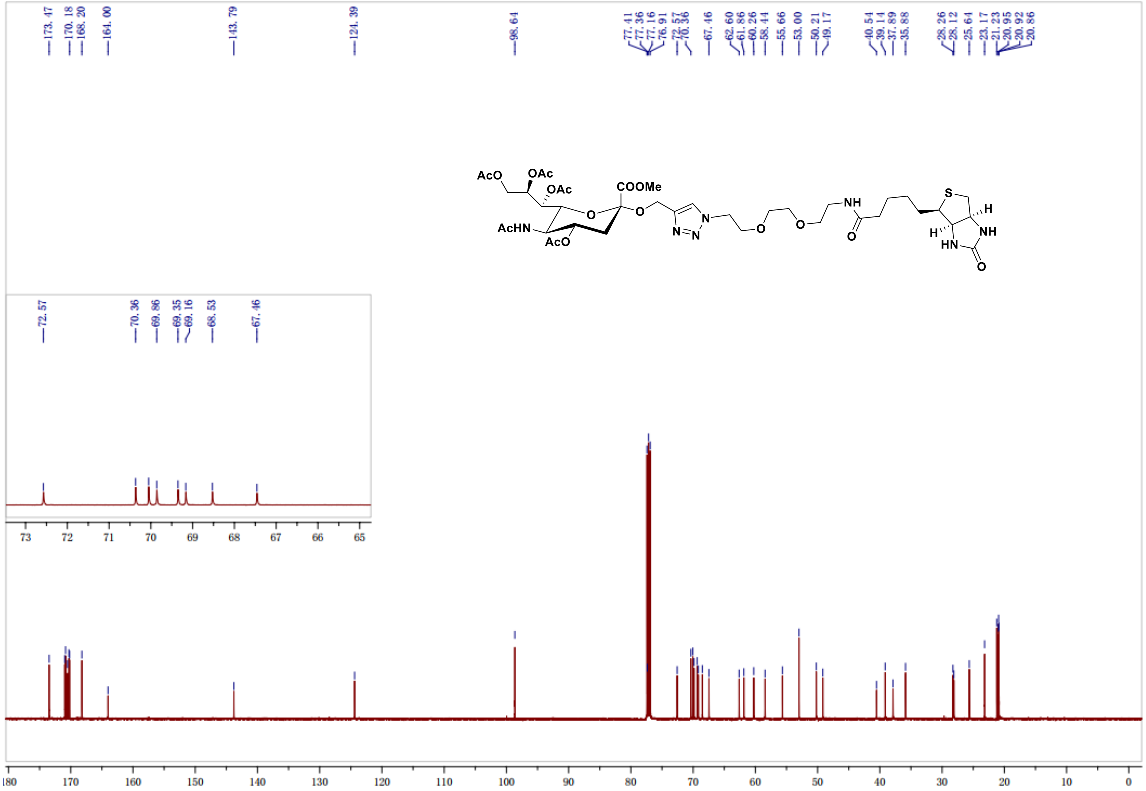

^{1}H NMR spectrum of **S2** ($CDCl_3$, 500 MHz)

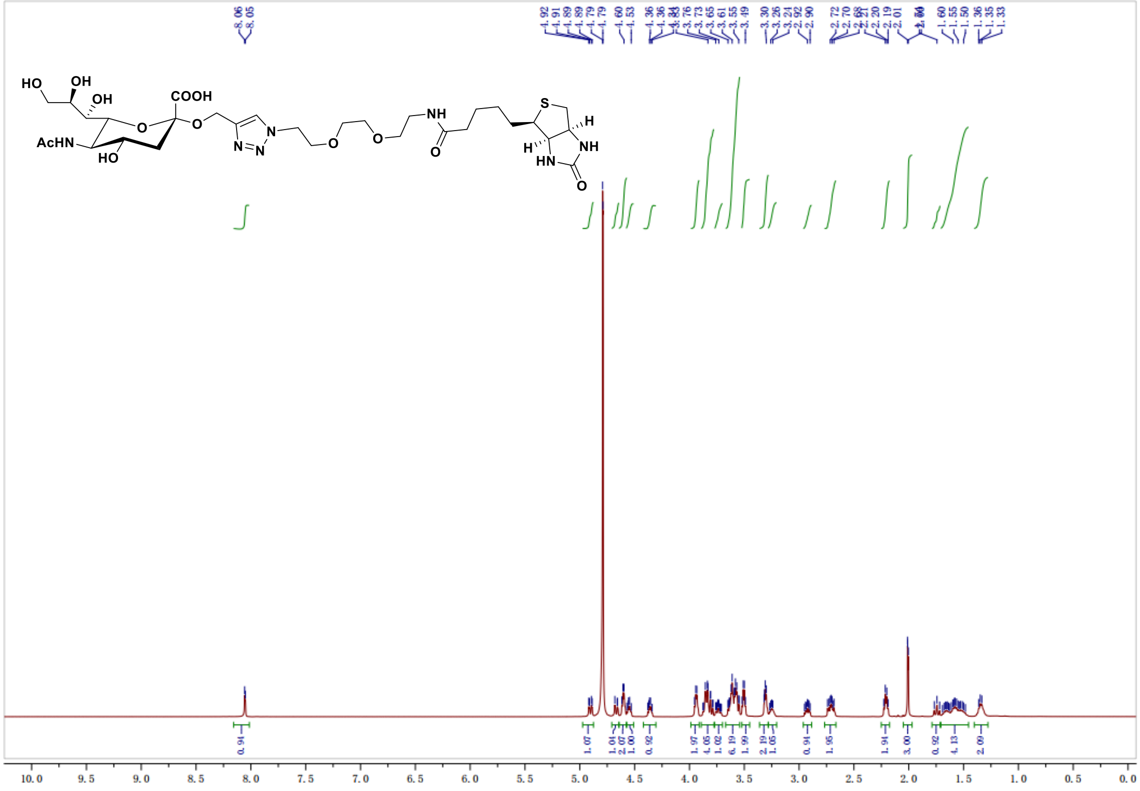

^{13}C NMR spectrum of **S2** ($CDCl_3$, 126 MHz)

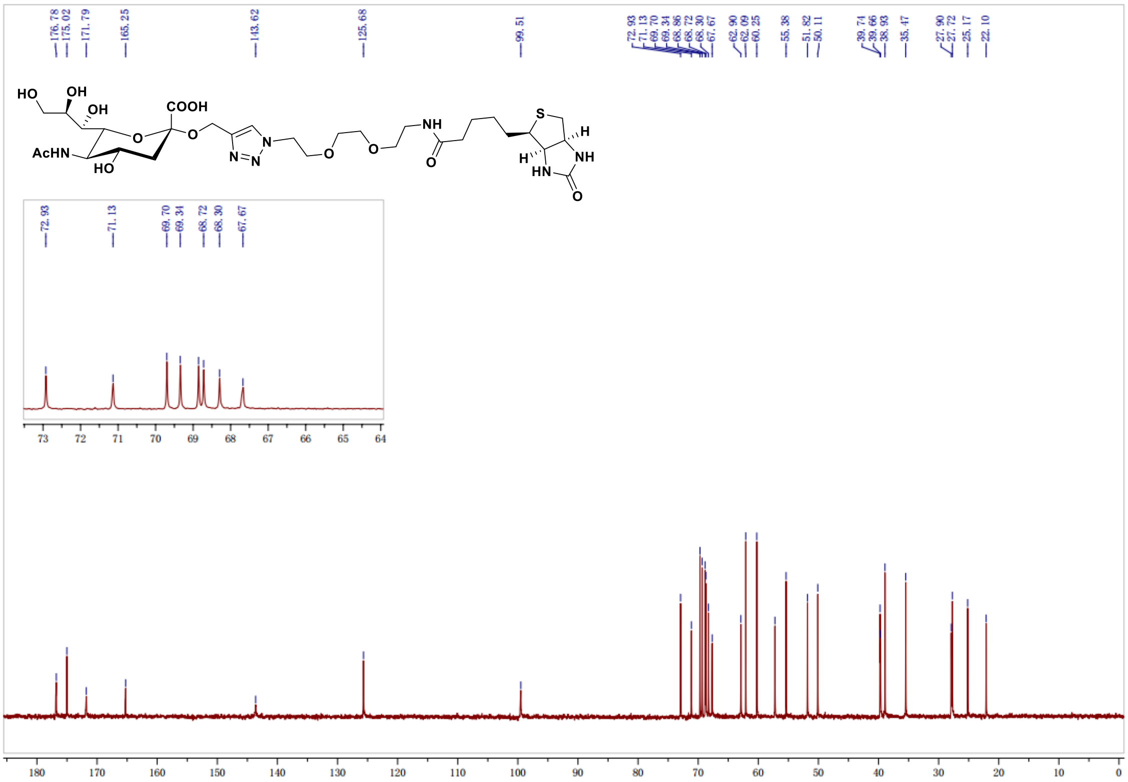

^{1}H NMR spectrum of **S3** (CD_3OD, 500 MHz)

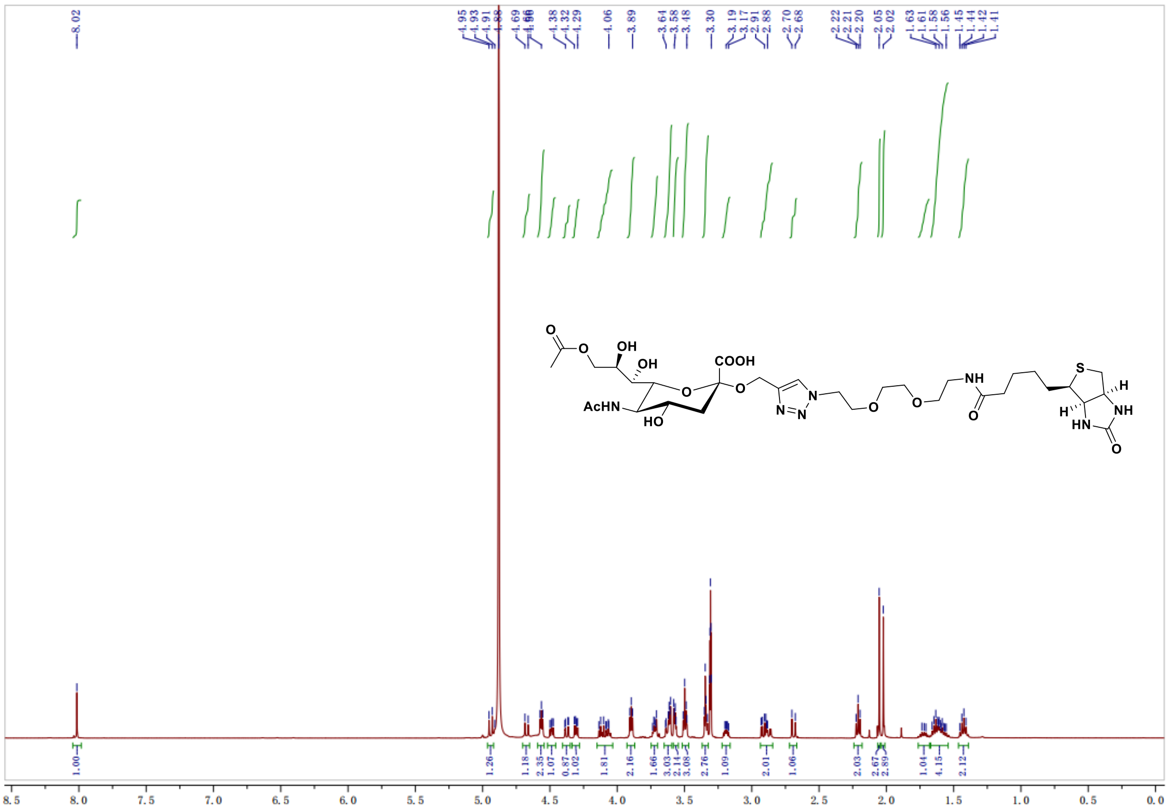

^{13}C NMR spectrum of **S3** (CD_3OD, 126 MHz)

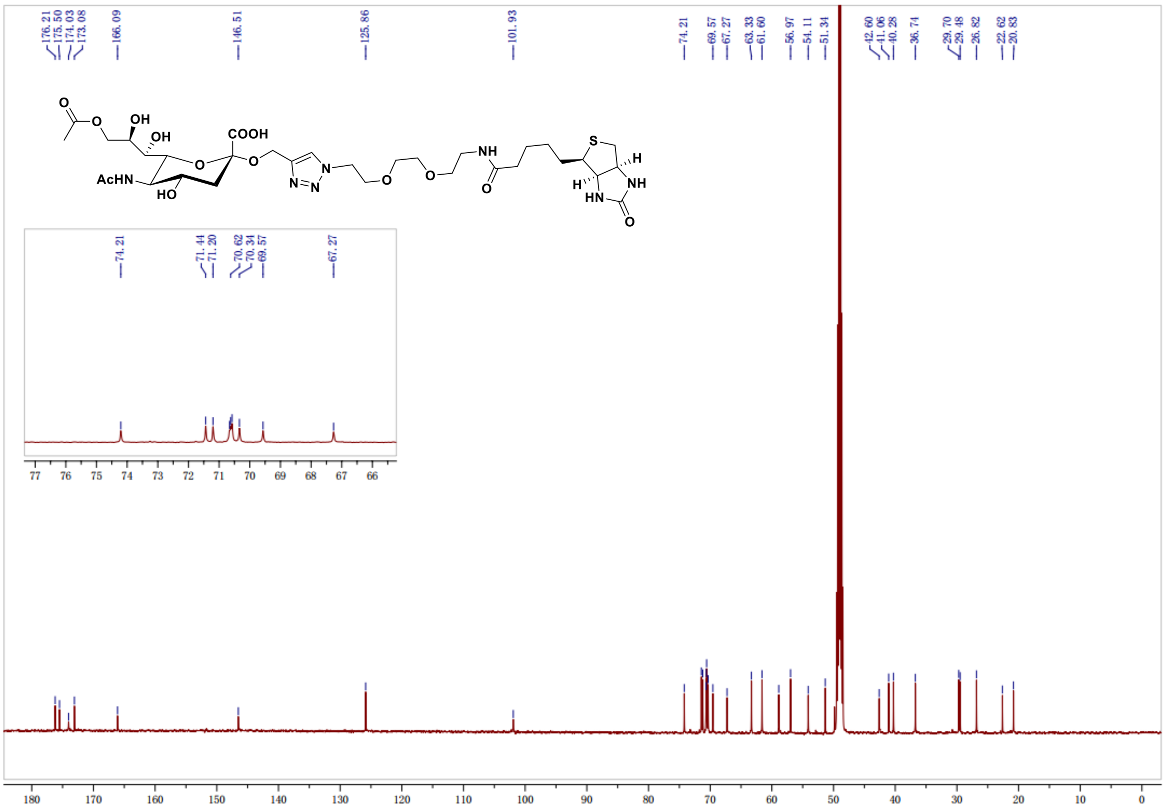

^1H -^1H NMR spectrum of **S3** (CD_3OD, 500 MHz)

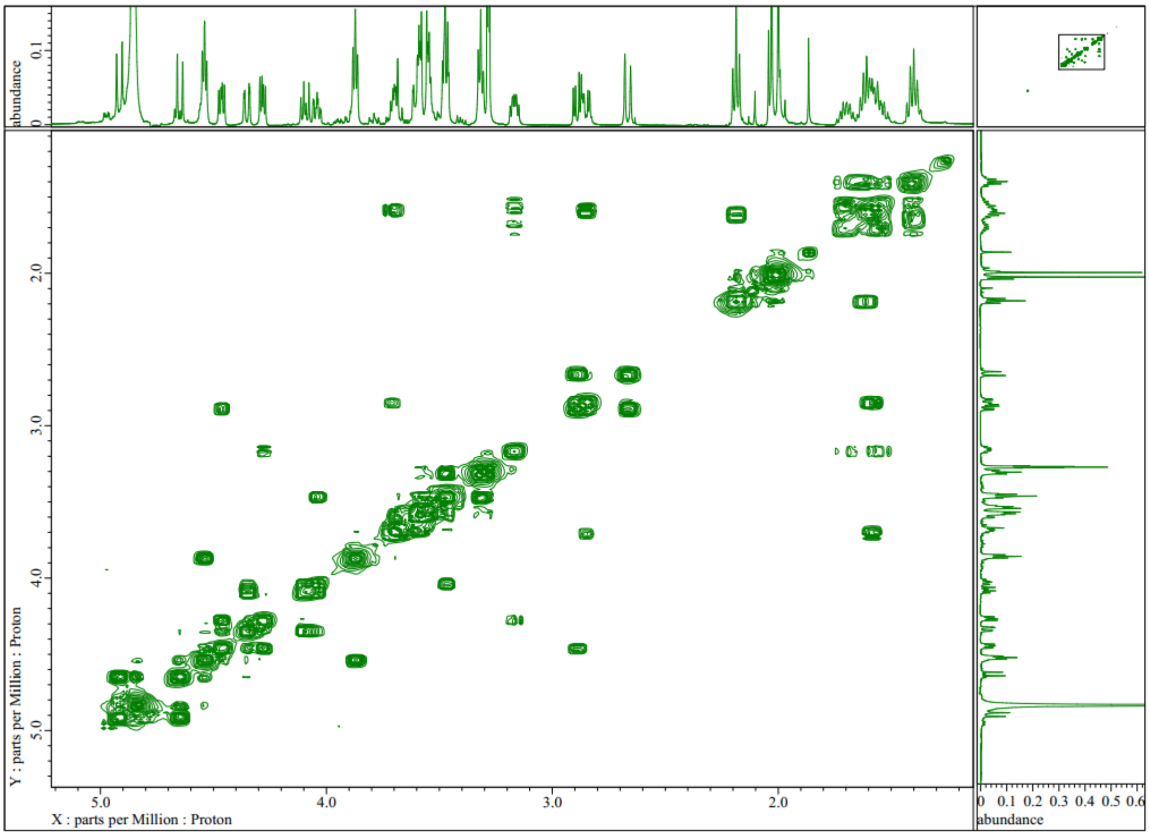

HMQC spectrum of **5** (CD_3OD, 500 MHz)

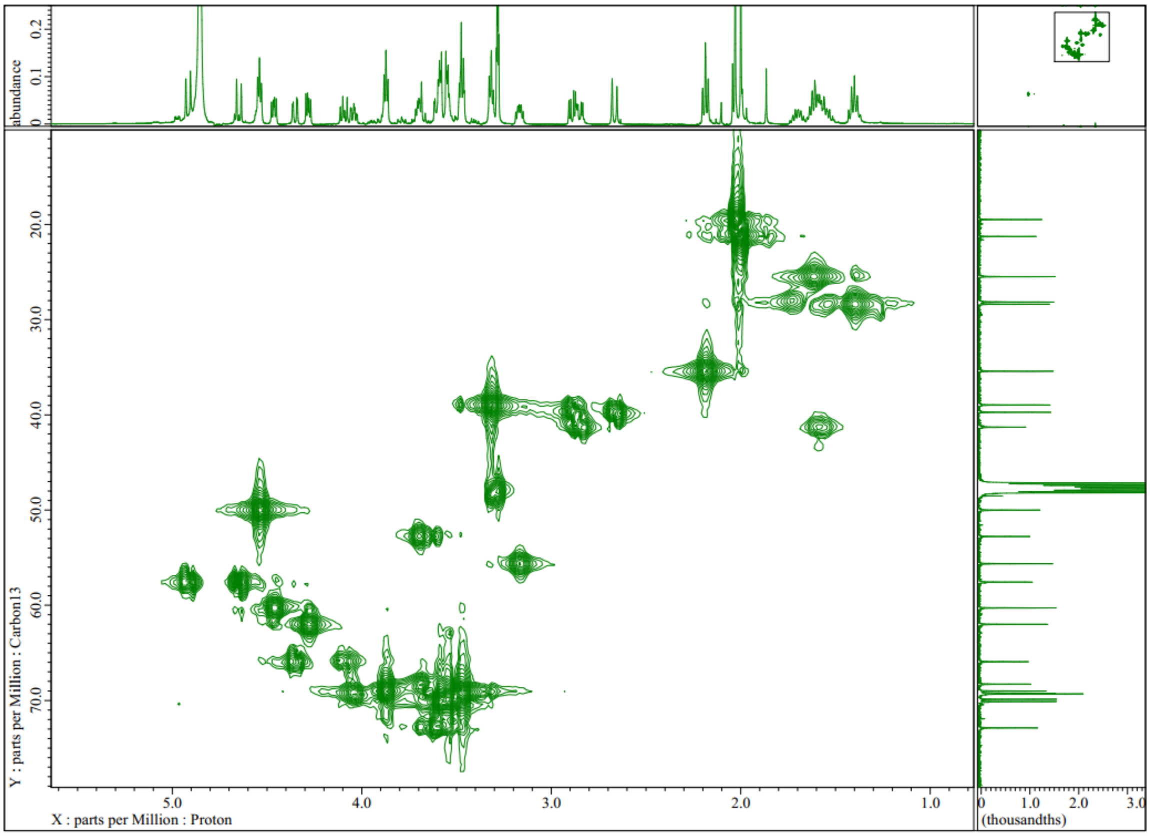

HMRS spectra

Compoun **S1**

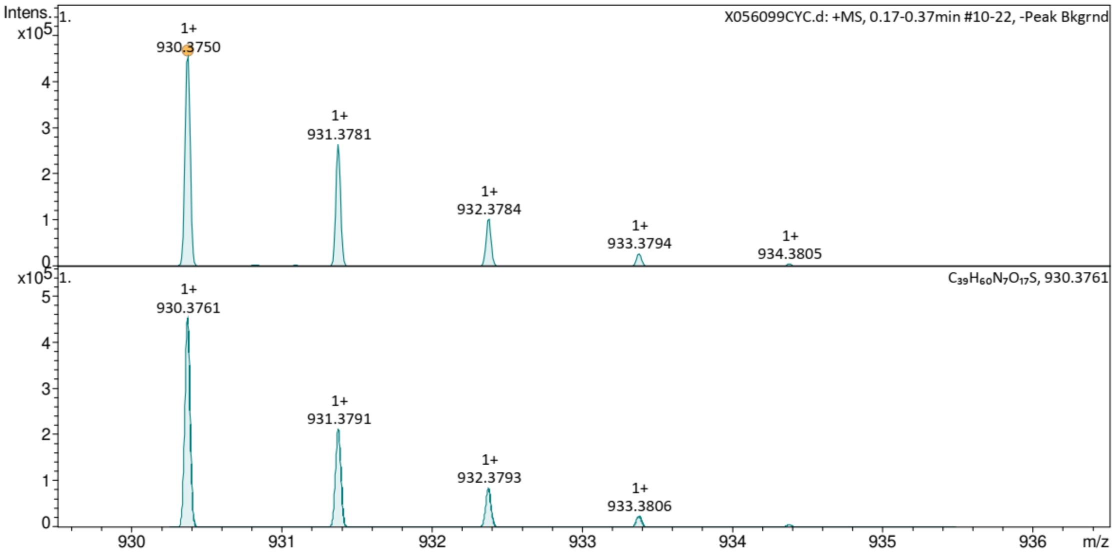

Compound S2

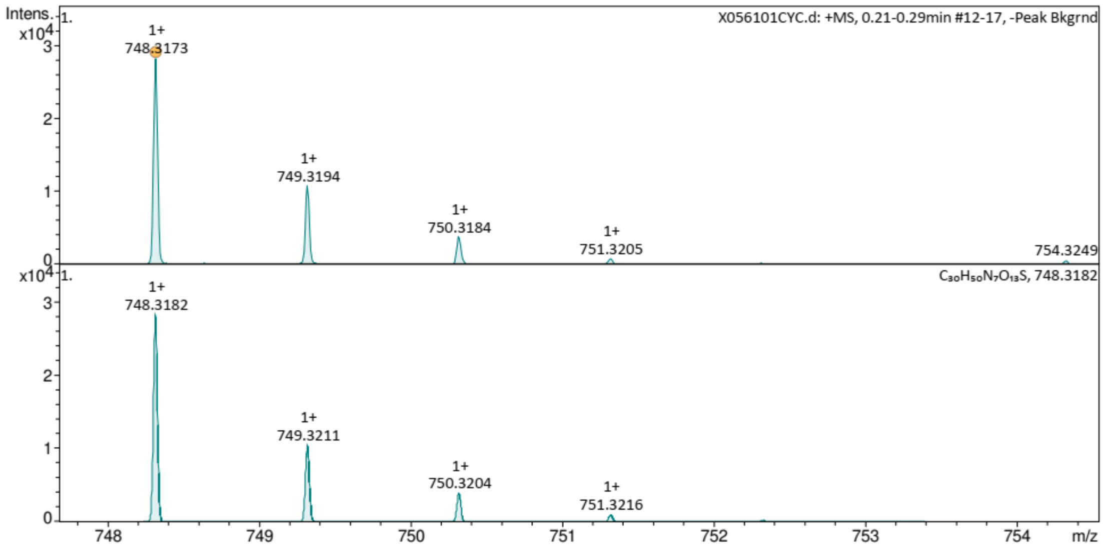

Compound S3

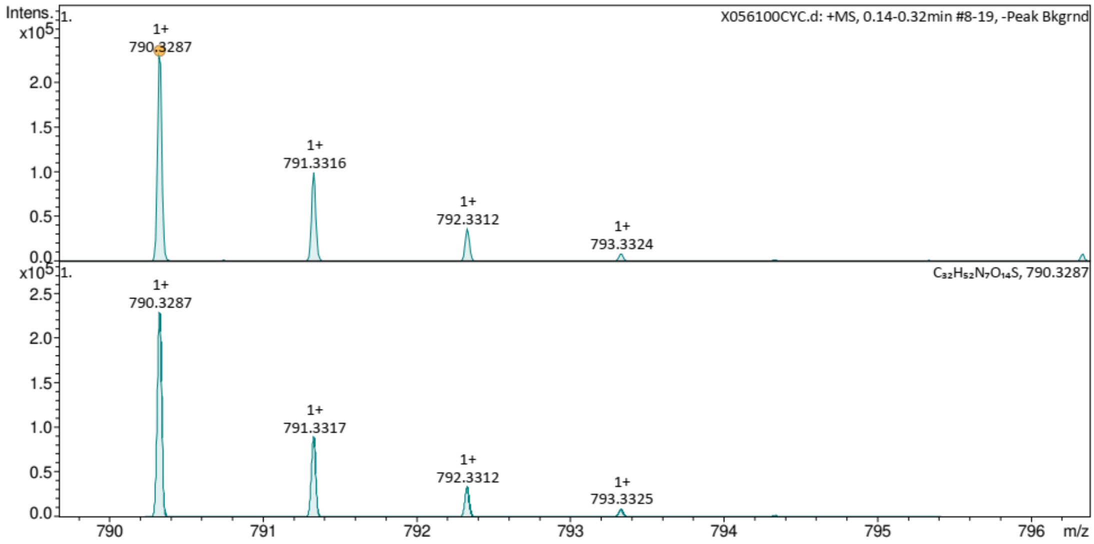

References

1 Nierengarten, I., Guerra, S., Holler, M., Nierengarten, J. F. & Deschenaux, R. Building liquid crystals from the 5-fold symmetrical pillar[5]arene core. *Chem. Commun.,* **48**, 8072-8074, doi:10.1039/c2cc33746k (2012).

2 Tikad, A. *et al.* Mechanistic Insight into Heptosyltransferase Inhibition by using Kdo Multivalent Glycoclusters. *Chem. Eur.J.,* **22**, 13147-13155, doi:10.1002/chem.201602190 (2016).

3 Liu, Y. *et al.* Protein-Framed Multi-Porphyrin Micelles for a Hybrid Natural–Artificial Light-Harvesting Nanosystem. *Angew. Chem. Int. Ed. Engl.,* **55**, 7952-7957 (2016).

4 Nierengarten, J. F. *et al.* Fullerene sugar balls. *Chem. Commun.,* **46**, 3860-3862, doi:10.1039/c0cc00034e (2010).

5 Ogura, H. *et al.* Synthesis of 9-O-acyl- and 4-O-acetyl-sialic acids. *Carbohydr. Res.* **167**, 77-86, doi:10.1016/0008-6215(87)80269-0 (1987).

6 Daskhan, G. C., Pifferi, C. & Renaudet, O. Synthesis of a New Series of Sialylated Homo-and Heterovalent Glycoclusters by using Orthogonal Ligations. *ChemistryOpen,* **5**, 477-484 (2016).

7 Jung, D., Maiti, S., Lee, J. H., Lee, J. H. & Kim, J. S. Rational design of biotin-disulfide-coumarin conjugates: a cancer targeted thiol probe and bioimaging. *Chem. Commun.,* **50**, 3044-3047, doi:10.1039/c3cc49790a (2014).

8 Gan, Z. & Roy, R. Sialoside clusters as potential ligands for siglecs (sialoadhesins). *Can. J. Chem.,* **80**, 908-916 (2002).
