# Supplementary material for: Multivalent 9-O-Acetylated-sialic acid glycoclusters as potent inhibitors for SARS-CoV-2 infection
Source: Nat Commun. 2022 May 10;13:2564. doi: 10.1038/s41467-022-30313-8 (PMC9091252; doi:10.1038/s41467-022-30313-8)
Supplement: Supplementary file 3 — Reporting Summary [file 41467_2022_30313_MOESM3_ESM.pdf]

nature portfolio

Corresponding author(s): David Alsteens
Stéphane P. Vincent

Last updated by author(s): Apr 7, 2022

Reporting Summary

Nature Portfolio wishes to improve the reproducibility of the work that we publish. This form provides structure for consistency and transparency in reporting. For further information on Nature Portfolio policies, see our Editorial Policies and the Editorial Policy Checklist.

Statistics

For all statistical analyses, confirm that the following items are present in the figure legend, table legend, main text, or Methods section.

n/a	Confirmed	
☐	☒	The exact sample size (*n*) for each experimental group/condition, given as a discrete number and unit of measurement
☐	☒	A statement on whether measurements were taken from distinct samples or whether the same sample was measured repeatedly
☐	☒	The statistical test(s) used AND whether they are one- or two-sided<br>*Only common tests should be described solely by name; describe more complex techniques in the Methods section.*
☒	☐	A description of all covariates tested
☒	☐	A description of any assumptions or corrections, such as tests of normality and adjustment for multiple comparisons
☐	☒	A full description of the statistical parameters including central tendency (e.g. means) or other basic estimates (e.g. regression coefficient) AND variation (e.g. standard deviation) or associated estimates of uncertainty (e.g. confidence intervals)
☐	☒	For null hypothesis testing, the test statistic (e.g. *F*, *t*, *r*) with confidence intervals, effect sizes, degrees of freedom and *P* value noted<br>*Give P values as exact values whenever suitable.*
☒	☐	For Bayesian analysis, information on the choice of priors and Markov chain Monte Carlo settings
☒	☐	For hierarchical and complex designs, identification of the appropriate level for tests and full reporting of outcomes
☒	☐	Estimates of effect sizes (e.g. Cohen's *d*, Pearson's *r*), indicating how they were calculated

Our web collection on statistics for biologists contains articles on many of the points above.

Software and code

Policy information about availability of computer code

Data collection	Nanoscope software v9.2, Force Robot Control Software v.6.1.144, Zen Blue 2.3 (blue edition), Bioimager device (Amersham Typhoon v2.0.0.6)
Data analysis	Nanoscope analysis software (v1.9, Bruker), Origin Software (OriginLab, version 2019), JPK Data Analysis software (JPK, v7.1.14+), ImageJ (v1.52e)

For manuscripts utilizing custom algorithms or software that are central to the research but not yet described in published literature, software must be made available to editors and reviewers. We strongly encourage code deposition in a community repository (e.g. GitHub). See the Nature Portfolio guidelines for submitting code & software for further information.

Data

Policy information about availability of data

All manuscripts must include a data availability statement. This statement should provide the following information, where applicable:

- Accession codes, unique identifiers, or web links for publicly available datasets
- A description of any restrictions on data availability
- For clinical datasets or third party data, please ensure that the statement adheres to our policy

The Source data underlying Figs 2c,e,f,i; 3d,h,k; 5; 6 are provided as a Source Data file. The source data for the protein structure in Fig. 1 is available at the RCSB protein data base under the reference 6VXX.

Field-specific reporting

Please select the one below that is the best fit for your research. If you are not sure, read the appropriate sections before making your selection.

☒ Life sciences ☐ Behavioural & social sciences ☐ Ecological, evolutionary & environmental sciences

For a reference copy of the document with all sections, see nature.com/documents/nr-reporting-summary-flat.pdf

Life sciences study design

All studies must disclose on these points even when the disclosure is negative.

Sample size	Typically, more than one hundred data points are needed for single-molecule experiments. Here we are always above this value. Multiple replicates (as stated in the legends of the figures) were tested. No statistical methods were used to predetermine the sample size. The sample sizes were determined to be adequate based on the cibsistency. This methods has been used by previous studies with similar experiments (Alsteens et al., Nature Methods 2015; Alsteens et al. Nature Nanotechnology 2017; Delguste et al., Sciences Adv. 2018). Sample sizes for each experiment are stated in the figure legends.
Data exclusions	No data was excluded from the analysis.
Replication	The experiments were sucessfully replicated at least 3-5 times.
Randomization	All data collection was performed in radom order for different experimental replicates. For microscopy imaging and probing experiments, multiple fields were randomly acquired for all experiments.
Blinding	The investigators were not blinded during experiments and data collection. Data were analyzed by a second researcher.

Reporting for specific materials, systems and methods

We require information from authors about some types of materials, experimental systems and methods used in many studies. Here, indicate whether each material, system or method listed is relevant to your study. If you are not sure if a list item applies to your research, read the appropriate section before selecting a response.

Materials & experimental systems

n/a	Involved in the study	
☐	☒	Antibodies
☐	☒	Eukaryotic cell lines
☒	☐	Palaeontology and archaeology
☒	☐	Animals and other organisms
☒	☐	Human research participants
☒	☐	Clinical data
☒	☐	Dual use research of concern

Methods

n/a	Involved in the study	
☒	☐	ChIP-seq
☒	☐	Flow cytometry
☒	☐	MRI-based neuroimaging

Antibodies

Antibodies used	VSV-G antibody provided Part Gert Zimmer
Validation	The antibody were validated using ELISA and cited in multiple publications.

Eukaryotic cell lines

Policy information about cell lines

Cell line source(s)	Lec2 (ATCC® CRL-1736); CHO cells (ATCC® CCL-61); A-549 (ATCC® CCL-185); HEK-293T (ATCC® CRL-3216), Vero E6 cells (ATCC® CRL-1586)
Authentication	The cells obtained from ATCC were authenticated prior to purchase using STR profiling. The replicates of each experiment were conducted with cells from different passages, and no significant difference was observed among these replicates.
Mycoplasma contamination	We did not see any indicators of mycoplasma contamination.
Commonly misidentified lines (See ICLAC register)	The cell lines used are not listed as commonly misidentified in the ICLAC register.
